# Systemic inflammation after stroke: implications for post‐stroke comorbidities

**DOI:** 10.15252/emmm.202216269

**Published:** 2022-08-15

**Authors:** Alba Simats, Arthur Liesz

**Affiliations:** ^1^ Institute for Stroke and Dementia Research (ISD), University Hospital LMU Munich Munich Germany; ^2^ Munich Cluster of Systems Neurology (SyNergy) Munich Germany

**Keywords:** inflammation, long‐term outcome, post‐stroke comorbidities, stroke, systemic immunity, Cardiovascular System, Immunology, Neuroscience

## Abstract

Immunological mechanisms have come into the focus of current translational stroke research, and the modulation of neuroinflammatory pathways has been identified as a promising therapeutic approach to protect the ischemic brain. However, stroke not only induces a local neuroinflammatory response but also has a profound impact on systemic immunity. In this review, we will summarize the consequences of ischemic stroke on systemic immunity at all stages of the disease, from onset to long‐term outcome, and discuss underlying mechanisms of systemic brain‐immune communication. Furthermore, since stroke commonly occurs in patients with multiple comorbidities, we will also overview the current understanding of the potential role of systemic immunity in common stroke‐related comorbidities, such as cardiac dysfunction, atherosclerosis, diabetes, and infections. Finally, we will highlight how targeting systemic immunity after stroke could improve long‐term outcomes and alleviate comorbidities of stroke patients.

## Graphical abstract

This Review discusses the impact of ischemic stroke on systemic immunity, its interaction with common comorbidities, and the underlying mechanisms of systemic brain‐immune communication.

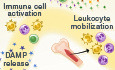

**Glossary**
Atherosclerosis
: The clogging or hardening of arteries caused by atheromatous plaques (accumulations of lipid deposits, usually cholesterol).
Cardioembolic stroke
: A subtype of ischemic stroke caused by a blood clot that forms in the heart and travels through the bloodstream to the brain.
Comorbidity
: Any coexisting health condition in addition to a primary disease.
Cryptogenic stroke
: A subtype of ischemic stroke of unknown etiology (clinically undetermined cause).
Cytokines
: Soluble mediators (peptide or protein) that are used for cell–cell communication between immunocompetent cells; directs their cell function and proliferation.
Diabetes
: Disorder of the carbohydrate metabolism characterized by impaired ability of the body to produce or respond to insulin and thereby maintain physiological blood glucose levels.
Endovascular therapy
: Therapeutic approach for cardiovascular diseases that uses minimally invasive, catheter‐based procedures to remove obstructive clots from inside the artery.
Intraparenchymal hemorrhage
: A type of bleeding that occurs within the brain parenchyma.
Ischemic stroke
: The most common type of vascular brain injury; brain lesion is caused by occlusion of a brain‐supplying artery.
Lymphopenia/lymphocytopenia
: A condition of pathologically reduced blood lymphocyte counts.
Recanalization
: Spontaneous or induced restoration of the blood flow in an occluded vessel or artery.
Subarachnoid hemorrhage
: A type of bleeding that occurs in the subarachnoid space, the space between the membranous layers of the arachnoid mater and the pia mater surrounding the brain.
Sympathetic hyperactivity
: Increased activity of the sympathetic nervous system.

## Introduction

Stroke is a major public health concern with a vast socioeconomic burden (Virani *et al*, 2021). Stroke is also the second leading cause of death and a leading cause of long‐term disability worldwide (Feigin *et al*, 2021). Despite enormous improvements on diagnosis and therapeutic strategies, the number of incident strokes is expected to more than double by 2050, and the prevalence of long‐term disabilities after stroke is anticipated to equally increase due to demographic changes and the growing number of stroke survivors (Howard & Goff, 2012). Stroke can be of ischemic or hemorrhagic nature. Approximately 70% of strokes are ischemic strokes, caused by the occlusion of a major cerebral artery, whereas others are hemorrhagic strokes, characterized by bleedings in the brain substance (intraparenchymal hemorrhages) or the subarachnoid space (SAHs). This review specifically deals with ischemic stroke.

At present, treatment interventions for ischemic stroke are limited to acute revascularization strategies, via the administration of thrombolytic agents or through endovascular therapy (catheter‐based mechanical thrombectomy). Both types of therapies aim at restoring blood flow to the hypo‐perfused brain tissue and need to be applied to patients as early as possible after stroke onset. This narrow therapeutic time window and several medical contraindications seriously reduce the number of stroke patients who currently can benefit from these recanalization therapies; hence, new treatment strategies are still urgently needed. As alternative methods to treat ischemic stroke, many neuroprotective agents have been evaluated during past decades to minimize the destructive pathophysiology of stroke and protect the ischemic brain (Patel & McMullen, 2017). The vast majority of these treatments target factors participating in the very early processes of ischemic cell death in the affected brain area, which beyond brain cells also comprises a heterogeneous and complex vascular network (Schaeffer & Iadecola, 2021). Compared to these acute neuroprotective approaches, much less attention has been given to other biological processes that have emerged in recent years as critical pathophysiological processes of stroke, such as systemic inflammation. Systemic poststroke inflammation has been identified as an important determinant of acute and long‐term prognosis of stroke patients (Dziedzic, 2015; Anrather & Iadecola, 2016). As such, systemic inflammation after stroke has become a novel target for translational research. Some first clinical trials aiming to tackle inflammation to minimize patients' functional disabilities and also prevent secondary comorbidities have been already conducted (Iadecola *et al*, 2020).

On this basis, this review focuses on the consequences of stroke on different branches of the systemic immune response. We will review current knowledge of the systemic changes of the immune system after ischemic stroke and how they might impact on poststroke acquired or pre‐existing comorbidities. We will mainly focus on modifiable comorbidities including infections, cardiovascular events, atherosclerosis and diabetes—although other no‐modifiable factors such as age and sex can also modulate the inflammatory response to stroke and determine the impact of inflammation on the outcome. Our review will also highlight the increasing and indisputable importance of poststroke systemic immunity on patients' long‐term outcome and its potential therapeutic value for the prevention of poststroke adverse events.

## Systemic poststroke inflammation: a multi‐phasic cascade

Ischemic stroke is caused by an abrupt loss of local cerebral blood flow in the brain. The lack of oxygen and nutrient supply evolves in a complex chain of biochemical and molecular events, leading to ischemic pan‐necrosis of the affected brain tissue (Iadecola *et al*, 2020). This ischemic brain injury results in the release of immunoactive molecules (damage‐associated molecular patterns, DAMP) that, on one side, locally activate immunocompetent cells, such as microglia and astrocytes. On the other side, DAMP release to the blood circulation can promote the recruitment of circulating immune cells to the brain and activate a complex peripheral immune response to stroke (Iadecola *et al*, 2020).

**Figure 1:**
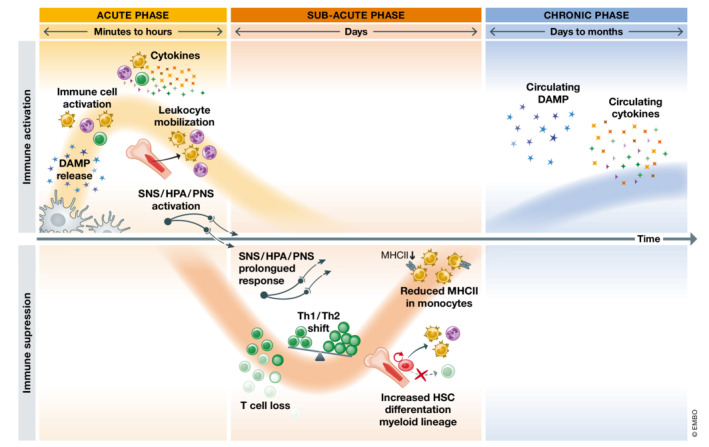
Main hallmarks of poststroke systemic inflammation over time The peripheral immune response to stroke is initiated within minutes after stroke onset. DAMP are originated from dying or stressed cells within the ischemic brain or actively secreted by immune cells upon activation. Circulating DAMPs activate peripheral immune cells and provoke a massive expression and release of pro‐inflammatory cytokines into the bloodstream. Within the acute phase, stroke also induces the mobilization of more leukocytes from the spleen and the bone marrow as well as the activation of neurogenic pathways. In the subacute phase, within hours to days after stroke onset, a state of immunosuppression is triggered. The prolonged overactivation of neurogenic pathways as well as DAMP and other pro‐inflammatory mediators acutely released after stroke gradually induce lymphopenia due to massive cell death and the pronounced bias towards the monocyte differentiation pathway in bone marrow hematopoiesis. The is also a disbalance between Type 1 (Th1) and Type 2 (Th2) helper T cells and circulating monocytes are less capable of providing costimulatory signals. Later in time, a long‐term phase compromising peripheral immunity is characterized by chronic and sustained high levels of DAMP and pro‐inflammatory cytokines.

During past decades, systemic immune changes after stroke were mainly studied in the context of subacute immunosuppression due to its association with the increased susceptibility to bacterial infections in stroke patients (Faura *et al*, 2021). More recent findings from numerous independent studies, however, have highlighted a previously less recognized multiphasic immune response to stroke, pointing out the importance of systemic inflammation throughout all stages of the disease, from onset to long‐term outcome. In the hyperacute phase after stroke, the peripheral immune system rapidly activates as a response to the stroke‐induced brain injury. This first acute systemic response is followed by a state of immunosuppression, which is characterized by loss and unresponsiveness of immune cells. Later in the chronic phase after stroke, a third and less well‐understood phase is characterized by a low‐grade sustained residual inflammation that might potentially impact on the long‐term outcome of stroke patients (Fig 1).

### Acute systemic inflammatory response

The peripheral immune response to stroke is initiated within minutes after stroke onset by DAMP originated from dying or stressed cells within the ischemic brain or actively secreted by macrophages and other immune cells (Muhammad *et al*, 2008; Schulze *et al*, 2013; Kunze *et al*, 2014; Richard *et al*, 2016; Schuhmann *et al*, 2021). Poststroke circulating DAMP comprise a diverse group of molecules. During past decades, converging evidence has shown that DAMP levels rapidly increase in blood within the first hours after stroke onset, both in preclinical and clinical studies of ischemic stroke (Table EV1). For instance, this is the case of the high‐mobility group box 1 protein (HMGB1), a nuclear chaperone protein, and calprotectin (S100A8/A9), a heterodimeric complex of the S100 family of proteins (Kim *et al*, 2006, 2018; Schulze *et al*, 2013; Liesz *et al*, 2015; Tsukagawa *et al*, 2017; Schuhmann *et al*, 2021; Roth *et al*, 2021a; Denorme *et al*, 2022). Similarly, peroxiredoxins (Prx), including Prx‐1 and Prx‐5, which function as peroxide scavengers under physiological conditions, and heat shock proteins (Hsp), that encompass a large family of chaperones, are also known to become danger signals that propagate inflammation within the first 24 h after stroke onset (Gruden *et al*, 2013; Kunze *et al*, 2014; Richard *et al*, 2016). More recently, circulating levels of cell‐free DNA (cfDNA), which increase in stroke patients as early as 4.5 h after stroke onset (Tsai *et al*, 2011; O'Connell *et al*, 2017; Roth *et al*, 2021a), have also been characterized as another type of circulating DAMP involved in the post‐stroke inflammatory response.

The exact mechanisms whereby the injured brain sends out these first DAMP signals to trigger acute systemic inflammation still remain unclear. The primary efflux route for the transiting of these molecules from brain to blood is thought to be the passive diffusion across the disrupted blood–brain‐barrier (BBB). The proteolytic degradation of the tight junction protein complexes and basement membranes, the loss of vascular cells and the increase in transcytosis of leukocytes are key factors contributing to the opening of the BBB, and thus facilitating a passive exit of brain‐derived DAMP as early as within hours after stroke onset (Abdullahi *et al*, 2018; Li *et al*, 2018). Besides this passive diffusion, the glymphatic and meningeal lymphatic systems have been also recently described as complementary candidate routes for the clearance of immune cells and macromolecules out of the brain (Bower & Hogan, 2018; Rasmussen *et al*, 2018; Lv *et al*, 2021). In this regard, clinical evidence also supports the involvement of these newly described pathways in the context of ischemic stroke, since neuronal antigens, including microtubule‐associated protein‐2 (MAP‐2) and N‐methyl D‐aspartate (NMDA) receptor subunit NR‐2A have been also observed in the draining lymph nodes of patients within the subacute phase after ischemic stroke (mean collection time: 76 h ± 34 h) (Planas *et al*, 2012).

**Figure 2:**
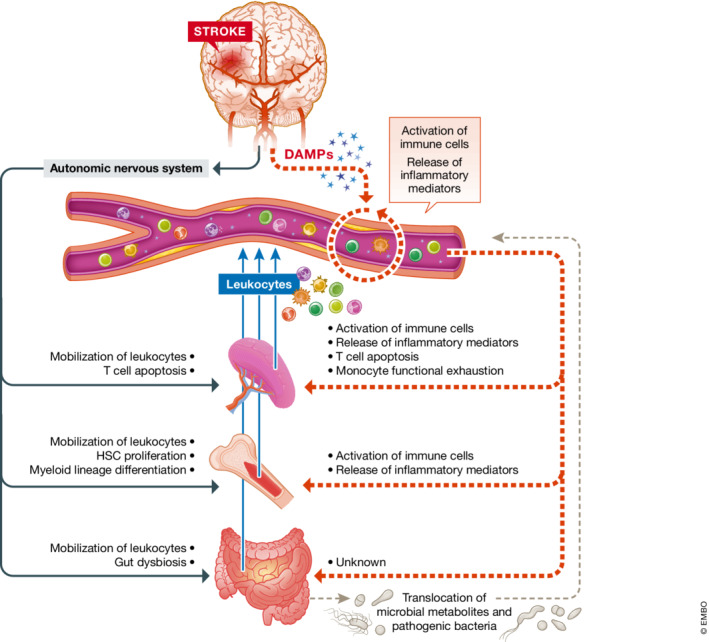
Mechanisms contributing to post‐stroke immune system activation Key effects triggered by the activation of the autonomic nervous system (black) or the circulating DAMP (red) in blood, spleen, bone marrow and gut, primary organs of the immune system.

DAMP are also produced and released into circulating by other cell types that rapidly become compromised and activated after stroke, including endothelial cells and distinct types of immune cells. For instance, HMGB1 or Prx‐2 are known to be released by monocytes and macrophages (Andersson & Tracey, 2011; Salzano *et al*, 2014), as well as by activated endothelial cells (Kang *et al*, 2014). Similarly, activated neutrophils release reactive oxygen and nitrogen species, myeloperoxidase (MPO), and neutrophil extracellular traps (NETs), scaffolds of nuclear or mitochondrial cfDNA surrounded by proteases and cytotoxic histones, among others (Garcia‐Bonilla *et al*, 2014; Kim *et al*, 2019). All these DAMP rapidly trigger a massive release of pro‐inflammatory cytokines by activated immune cells, another main hallmark of this first phase of the peripheral immune response to stroke (Fig 2). Circulating DAMP are recognized by pattern recognition receptors (PRRs) such as toll‐like receptors (TLRs) or the receptor for advanced glycation end products (RAGE), expressed by diverse immune cell subpopulations. Signaling through these PRRs activates diverse downstream signaling pathways, including the nuclear factor (NF)‐κB, mitogen‐activated protein kinase (MAPK), interferon regulatory factors (IRF) or the inflammasome signaling pathways (Roth *et al*, 2018; Alishahi *et al*, 2019; Li & Wu, 2021). The activation of these pathways directly leads to an increased expression of pro‐inflammatory cytokines. In experimental stroke, this early up‐regulation of pro‐inflammatory mediators has been well‐documented in the blood but also in lymphatic organs, including the lymph nodes and the spleen (Offner *et al*, 2006a; Esposito *et al*, 2019). For instance, several preclinical studies documented that the hyperacute (<4 h) peripheral inflammatory response in mice is dominated by the upregulation of pro‐inflammatory cytokines such as IL‐1β, IL‐6, TNF‐α, and IFN‐γ, as well as pro‐inflammatory chemokines, including C‐C motif chemokine ligand (CXCL)‐1 and CXCL‐2 (Offner *et al*, 2006a; Seifert *et al*, 2012; Esposito *et al*, 2019). Several of these upregulated pro‐inflammatory cytokines are key mediators of the stroke‐induced BBB breakdown and their levels have been associated with increased vascular permeability and larger infarct sizes (Yang *et al*, 2019). Similarly, high systemic levels of such pro‐inflammatory cytokines (IL‐6, TNF‐α and IL‐1β) have been directly related to cytokine‐induced sickness behavior after experimental stroke (Roth *et al*, 2021b).

In humans, it is now clear that within the first 12 h after stroke, there is also a pronounced increase in the circulating levels of such pro‐inflammatory cytokines, including TNF‐α and IL‐6 (Zaremba & Losy, 2001; Waje‐Andreassen *et al*, 2005; Basic Kes *et al*, 2008; Tuttolomondo *et al*, 2009). In addition, blood levels of these cytokines have been also positively correlated with stroke severity and unfavorable prognosis of stroke patients (Basic Kes *et al*, 2008; Aref *et al*, 2020).

Beyond the massive release of pro‐inflammatory cytokines, leukocytes are also rapidly mobilized from the spleen and the bone marrow, two major reservoirs of immune cells (Seifert *et al*, 2012; Courties *et al*, 2015). These cell reservoirs are however limited, and rapidly exhaust within hours after stroke. Thus, at this early time‐point after stroke, the bone marrow also increases hematopoiesis to replenish the pool and meet the demand of leukocytes in circulation (Courties *et al*, 2015). Mechanistically, the stroke‐induced activation of neurogenic pathways, including the sympathetic innervation, hypothalamic–pituitary–adrenal (HPA) axis and parasympathetic innervation, play a major role in the release of immune cells from these two peripheral reservoirs (Fig 2). It is now known that the observed increase in circulation of norepinephrine and epinephrine levels acutely after stroke contributes to spleen shrinkage and massive exiting of immune cell populations from this organ (Ajmo *et al*, 2009). Similarly, in the bone marrow, the early activation of sympathetic innervation is observed by an abrupt increase in the levels of tyrosine hydroxylase and norepinephrine within the first day after stroke (Courties *et al*, 2015). The early activation of hematopoietic stem cells proliferation and differentiation has been attributed to this post‐stroke increased sympathetic tone. Likewise, in bone marrow mesenchymal stromal cells, activation of β3‐adrenergic receptors further results in a downregulation of homeostatic and cell retention factors, including IL‐7, C‐X‐C motif chemokine 12 (also known as stromal cell‐derived factor 1), VCAM‐1 and angiopoietin‐1, which enables the exiting of leukocytes into the bloodstream (Courties *et al*, 2015).

Recently, the gut and gastrointestinal tract has been described as an another important reservoir of immune cells, from which leukocytes are also mobilized to the circulation following stroke and even recruited to the ischemic brain (Benakis *et al*, 2016; Singh *et al*, 2016; Brea *et al*, 2021). In addition, increasing data also indicates that the intestinal microbiota also plays a role in modulating the phenotype of the immune cells within the acute inflammatory response to stroke (Benakis *et al*, 2016; Singh *et al*, 2016; Lee *et al*, 2020). Despite the exact mechanisms whereby the microbiome sense the ischemic brain injury and primes the post‐stroke inflammatory response are not fully understood, several studies have already described that neurohumoral signals generated by the ischemic brain perturb immune homeostasis in the gut and lead to changes in the gut microbiota composition (Benakis *et al*, 2016; Houlden *et al*, 2016; Singh *et al*, 2016). Moreover, stroke‐induced alterations in gut microbiome composition have been associated with worse stroke outcome, larger infarct volumes and poorer scores in functional tests (Benakis *et al*, 2016, 2020; Singh *et al*, 2016; Xu *et al*, 2021; Honarpisheh *et al*, 2022). This effect was largely attributed to polarization of circulating immune cells by the microbiome in the acute post‐stroke phase (Benakis *et al*, 2016; Singh *et al*, 2016; Lee *et al*, 2020). Likewise, poststroke dysbiosis has been also linked to reduced intestinal motility and intestinal barrier dysfunction, even leading to the translocation of intestinal bacteria into circulation and peripheral organs (Swidsinski *et al*, 2012; Yin *et al*, 2015; Houlden *et al*, 2016; Singh *et al*, 2016; Stanley *et al*, 2016; Liu *et al*, 2019). In this regard, the dissemination of selective bacterial species from the gut microbiota after the occurrence of stroke has been proposed to be a plausible source of poststroke infection, and might even potentially contribute further to the systemic pro‐inflammatory immune activation after stroke (Stanley *et al*, 2016). However, it is neither clear yet whether other factors also influence bacterial translocation after experimental stroke, nor whether these findings also occur in stroke patients.

Recent evidence suggests that activated endothelial and circulating innate immune cells after stroke could also promote immunothrombosis, the inflammation‐dependent activation of the coagulation system, and thromboinflammation, the aberrant and excessive activation of immunothrombosis (Stark & Massberg, 2021). Similar to immunothrombosis in response to systemic infections, evidence is accumulating that poststroke sterile inflammation and activation of innate immune cells, such as monocytes and neutrophils, lead to the activation of the coagulation cascade and promote thrombosis (Engelmann & Massberg, 2013; De Meyer *et al*, 2016). These two functional interdependent processes (inflammation and coagulation) have the ability to potentiate each other and together are known to aggravate ischemic stroke injury and contribute to secondary thrombotic complications of stroke, including recurrent strokes or myocardial infarctions.

### Systemic immunosuppression

The early activation of the immune system is rapidly followed by a state of systemic immunodepression. The most distinguished feature of this systemic immunosuppressive phase is the reduction in circulating T, B and NK cell counts. In this line, early studies on the immune profile of stroke patients already described profound peripheral lymphopenia as early as one day after ischemic stroke (Haeusler *et al*, 2008; Klehmet *et al*, 2009). In mice, reduced levels of T, B and NK cells, and T cell responsiveness have been similarly documented both in circulation and in the spleen and lymph nodes already within 12 h after experimental stroke (Prass *et al*, 2003). This reduction of lymphocyte counts partly results from a drastic apoptotic death of immune cell populations in spleen (Offner *et al*, 2006b), which is also reflected by a reduction of spleen size (Yan & Zhang, 2014; Chiu *et al*, 2016). In the bone marrow after stroke, a suppression of the lymphoid lineage progression, which contributes further to decreasing lymphocyte counts in circulation, and a subsequent bias toward the monocyte differentiation pathway is observed (Courties *et al*, 2015).

Another characteristic trait of this systemic immunosuppressive state is the shift in the Type 1 (Th1) to Type 2 (Th2) helper T cell ratio. This phenomenon represents the disbalance between proinflammatory and cellular (Th1) and anti‐inflammatory and humoral (Th2) predominant mechanisms and is reflected by an increase in the circulating levels of anti‐inflammatory cytokines, such as IL‐10 and IL‐4, and a consequent reduction of pro‐inflammatory factors, including IFN‐γ and TNF‐α, among others (Jiang *et al*, 2017).

During this immunosuppressive state, circulating monocytes are also less capable of providing costimulatory signals required for activating T cells. In mice, this monocytic loss of function after experimental stroke has even been characterized by a reduction in the expression of genes associated with macrophage activation status (MHC Class II genes) and pathogen recognition ability (TLR genes) (McCulloch *et al*, 2018). In humans, the stroke‐induced monocytic loss of function is reflected by a reduction in the expression of human leukocyte antigen D‐ related (HLA‐DR) and CD64 on monocytes and dendritic cells (Krishnan *et al*, 2021).

Mechanistically, the suppression of the immune response is thought to be partly caused by the prolonged overactivation of the sympathetic nervous system (SNS) (Fig 2). In the spleen, the activation of both α and β adrenergic receptors in splenocytes by circulating catecholamines, rather than a direct effect of the sympathetic neurotransmission via the splenic nerves, is thought to be one potential mechanism that triggers splenic atrophy and T cell apoptosis (Prass *et al*, 2003; Ajmo *et al*, 2009). In bone marrow, the activation of the SNS results in the upregulation of a specific subset of T cells (regulatory T cells, Tregs) which display immunosuppressive traits and have been strongly associated with poststroke lymphocytopenia and immunosuppression (Wang *et al*, 2015). Moreover, the overactivation of the sympathetic tone also results in a substantial increase in the expression of myeloid transcription factors, such as PU.1 (Courties *et al*, 2015). This concludes with a pronounced increase in the myeloid lineage proliferation, a subsequent egress of myeloid cells to circulation and a suppression of the lymphoid lineage progression.

Beyond the SNS, the (over)activation of the hypothalamic pituitary adrenal (HPA) axis also has been proposed to participate in triggering immunosuppression after stroke. In a similar manner to catecholamines, high levels of glucocorticoids after stroke further contribute to lymphocyte apoptosis in spleen (Prass *et al*, 2003). Glucocorticoids also promote the production of anti‐inflammatory cytokines, like transforming growth factor β (TGF‐β), and suppress the secretion of pro‐inflammatory cytokines, such as IL‐1β, IL‐8, and TNF‐α. Involvement of both the SNS and HPA pathways in poststroke immunosuppression was further demonstrated in experiments blocking the respective pathways using propranolol and the glucocorticoid receptor inhibitor RU486, which resulted in a reduction of lymphocyte apoptosis and monocyte deactivation after experimental stroke (Prass *et al*, 2003). Recently, the glucocorticoid signaling pathway has been also involved in mediating brain‐bone marrow endocrine interaction, which negatively impact on lymphocyte production after stroke (Courties *et al*, 2019).

The parasympathetic nervous system (PNS) might also participate in promoting this immunosuppressive state. Although less well characterized than the SNS, the parasympathetic activity also increases after stroke (Engel *et al*, 2015). In response to infection or tissue damage, the cholinergic pathway is known to also act as an anti‐inflammatory protective mechanism to prevent the overactivation of the immune system (Rosas‐Ballina & Tracey, 2009). In particular, acetylcholine has been shown to inhibit macrophages and attenuate their release of pro‐inflammatory cytokines, including IL‐1β, IL‐6, and TNF‐α (Borovikova *et al*, 2000). Also, vagal nerve stimulation (VNS) has been shown to suppress the LPS‐induced increase in TNF‐α levels (Borovikova *et al*, 2000). Moreover, it has been also documented that a subpopulation of splenic T cells can also produce acetylcholine, which is required in the spleen for the inhibition of the massive cytokine secretion after VNS (Rosas‐Ballina *et al*, 2011). Although the connection between the cholinergic pathway and the spleen response to VNS is not fully understood, these findings suggest a possible role of the PNS in cell‐mediated immune suppression after stroke.

While several studies support the concept of the stress‐induced immunosuppression after stroke, clinical and experimental data reporting altered circulating levels of catecholamines after stroke are still controversial (Liesz *et al*, 2013; Mracsko *et al*, 2014). Hence, other mechanisms have been also been proposed to systemically contribute to lymphopenia and immunosuppression. In this regard, brain‐released DAMP and other pro‐inflammatory mediators such as cytokines in circulation early after stroke might also play a direct role in the subacute suppression of cellular immunity (Fig 2). For instance, the signaling pathway triggered by HMGB1 and its PRR RAGE, expressed on the surface of many immune cells, is known to participate in the bone marrow egress and splenic proliferation of immature monocytes, with lymphocyte‐suppressing traits (Liesz *et al*, 2015). Also, activation of the HMGB1‐RAGE axis has been shown to promote functional exhaustion of mature monocytes and lymphopenia, hallmarks of cell‐mediated immunosuppression (Liesz *et al*, 2015). High levels of circulating IL‐1β as a result of systemic inflammasome activation in the acute phase has been shown to induce the expression of Fas ligand (FasL). This is a ligand to the death receptor Fas (CD95), consequently resulting in Fas‐dependent T cell apoptosis. This mechanism might already initiate cell death of lymphocytes in the very early phase after stroke, when catecholamine concentrations in circulation might not even be increased yet (Roth *et al*, 2021a). Likewise, another stress‐independent mechanism directly affecting immunocompetence after stroke via immune mechanisms is known to be the release of arginase I (Arg1) from activated neutrophils. Arg1‐release from neutrophil granules has been associated with T‐cell dysfunction following a number of diverse pathologies, including ischemic stroke (Sippel *et al*, 2015).

### Chronic systemic inflammation after stroke

Little is known about the systemic immune state beyond the well‐described immunosuppression phase. Most clinical studies that do evaluate long‐term consequences of stroke are mainly centered on clinical data on patients' disabilities, functional outcome, and the development of secondary comorbidities and recurrent events, with limited records on molecular and biochemical data in these patient cohorts. Yet, few retrospective clinical studies have provided evidence that pro‐inflammatory mediators such as IL‐6 and IL‐1β remained elevated in circulation even 3 months after stroke onset (Liesz *et al*, 2013; Stanne *et al*, 2022). Levels of such cytokines showed a more pronounced increase in large strokes compared to small strokes, as dichotomized by the median of the lesion volume in the ischemic stroke group (Liesz *et al*, 2013). Similarly, plasma HMGB1 levels were also found to be increased in ischemic stroke patients from 24 h up to 90 days after stroke (Schulze *et al*, 2013; Roth *et al*, 2018). HMGB1 levels were also higher in patients with severe strokes (NIHSS ≥17), indicating an important role of stroke severity and volume on the magnitude of systemic immune alteration long‐term after stroke. Serum levels of monocyte chemoattractant protein (MCP)‐1 and circulating C‐reactive protein (CRP) have been also shown to persist at high levels in stroke patients at the 3‐month follow‐up compared to baseline (Garlichs *et al*, 2003; Ladenvall *et al*, 2006). Interestingly, blood CRP levels differ between stroke subtypes, since large‐vessel strokes had higher CRP levels at follow‐up compared with all other stroke subtypes, including small‐vessel strokes, cardioembolic strokes, cryptogenic strokes, and others (Ladenvall *et al*, 2006). More recent findings also reported a higher frequency of activated human leukocyte antigen (HLA^−^ DR^+^) cells in blood 2 months after ischemic stroke (Roth *et al*, 2018), and found that circulating IL‐4 and IFN‐γ levels persisted elevated up to 3 months poststroke, regardless of stroke etiology (Holmegaard *et al*, 2021). Hence, whether post‐stroke chronic inflammation could be influenced by the infarct topography and/or the co‐existence of any other comorbidity still needs to be elucidated.

Beyond stroke, this chronic inflammatory milieu has been also characterized in patients suffering from other acute brain lesions, including traumatic brain injury (TBI). Plasma IL‐6 levels were found to be substantially increased in patients 6 months after mild TBI, compared to orthopedic injury controls (Vedantam *et al*, 2021). Similarly, Chaban and colleagues found that other pro‐inflammatory cytokines, including IFN‐γ, IL‐8, MCP‐1 and macrophage inflammatory protein (MIP)‐1β, also remained increased for up to 1 year post‐TBI (Chaban *et al*, 2020).

Altogether, all these clinical findings suggest that after stroke, a third long‐term phase compromising peripheral immunity characterized by a chronic and sustained inflammatory milieu might also take place. Therefore, a complete understanding of these chronic systemic immune consequences of stroke and their modulation is of high translational relevance for ensuring better chronic patient outcomes.

## Stroke comorbidities: role of systemic immunity

**Figure 3:**
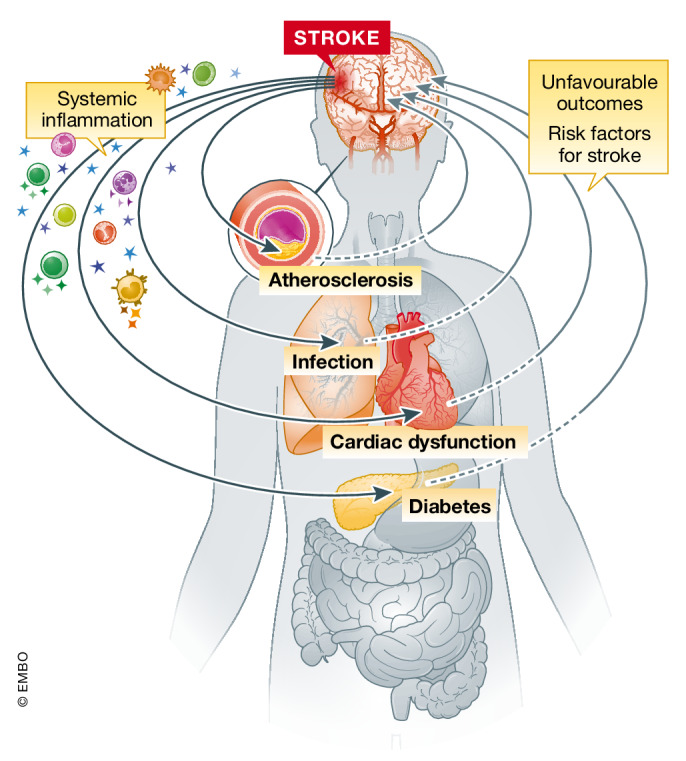
The stroke‐induced systemic inflammation represents a risk factor for the development of inflammation‐related comorbidities after stroke

The incidence for ischemic stroke is increasing, in part because the world's older population is dramatically growing. Besides age, the prevalence of multiple comorbidities and pre‐existing medical conditions is also increasing in both, the older and younger population (Katan & Luft, 2018). These adverse medical conditions further increase the incidence of stroke and highly worsen its outcome (Fig 3) (Gallacher *et al*, 2014). Indeed, stroke survivors especially in the elderly population commonly become multimorbid, predisposing two or more unfavorable medical coconditions. Some of these conditions, including heart diseases, atherosclerosis, hypertension, and diabetes mellitus, are pre‐existing or acquired disorders that share pathological mechanisms with stroke. In many cases, these conditions are as well risk factors for cardio‐ and cerebrovascular events and could even promote stroke recurrence, still a major complication of incident strokes. Other coexisting conditions might have also arisen from the primary stroke itself, such as post‐stroke infections and long‐term vascular dementia, among others (Gallacher *et al*, 2019). Interestingly, many of these pre‐existing or acquired comorbidities after stroke share inflammatory pathophysiological mechanisms among them, which might even potentiate or aggravate the development of other unfavorable medical conditions and worsen the long‐term outcome. Therefore, in this section, current literature on the association between stroke and its main comorbidities will be reviewed, with special emphasis on the subsequent systemic immune alterations as a key common factor in all these pathologies (Fig 3).

### Poststroke infections

Infections represent one of the main life‐threatening complications after stroke, accounting for approximately 30% of stroke patients (Westendorp *et al*, 2011). Most common poststroke infections are pneumonia and urinary tract infections (Bustamante *et al*, 2017). Despite advances in the field, effective clinical management of poststroke infections remains challenging. Current clinical strategies against stroke‐associated infections are based on treatment with broad‐spectrum antibiotics once infection has already developed and clinically diagnosed. Preventive antibiotic therapies before the development of clinical signs are also being evaluated as a manner to anticipate and prevent the onset of these fatal complications, although beneficial effects have not been yet demonstrated (Vermeij *et al*, 2018).

Several key factors whereby infections might develop after stroke and lead to an unfavorable outcome of stroke patients have been proposed so far. Patients' baseline characteristics, including age, functional disabilities, and dysphagia increase susceptibility to post‐stroke infections (Hoffmann *et al*, 2017). Also the use of invasive devices and clinical procedures, such as central venous and urinary catheters, secondary surgeries, or mechanical ventilation, might also be associated with a high incidence of infections (Ashour *et al*, 2016).

Beyond these obvious clinical factors, which are a common source of infections independent of stroke, the subacute immunosuppression after stroke discussed above has been tightly associated with increased susceptibility of stroke patients to infections. Alterations in the blood profile of several cytokines after stroke have been linked to an increased incidence of infections in stroke patients. This is the case for the anti‐inflammatory cytokine IL‐10, which has been found to be substantially increased at the onset of stroke in those patients who will develop poststroke infections within the first week after stroke and has even been proposed as a robust independent prognostic biomarker for these infectious complications (Chamorro *et al*, 2006; Ashour *et al*, 2016). Beyond IL‐10, the development of poststroke infections also correlates with lower TNF‐α levels and the consequent decrease in the TNF‐α/IL‐10 ratios at day 2 after stroke, this latest supporting a role of the Th1/Th2 shift in patients suffering from such complications (Chamorro *et al*, 2006). Other studies have also shown that patients who develop secondary infections after ischemic stroke show higher IL‐1β and IL‐6 blood concentrations on hospital admission and along the first 3 days after hospitalization, respectively (Wartenberg *et al*, 2011; Bustamante *et al*, 2014; Roth *et al*, 2021a). Elevated circulating monocyte counts within the first days after stroke are also associated with the development of post‐stroke infections (Urra *et al*, 2009). Moreover, HLA‐DR expression on monocytes from stroke patients was inversely correlated with the development of infections, and these “exhausted” monocytes of stroke patients also had a decreased capacity to release TNF‐α after stimulation. Indeed, this subpopulation of monocytes, primarily characterized by reduced levels of monocytic HLA‐DR expression, has become a robust independent factor to predict the occurrence of post‐stroke infections (Hoffmann *et al*, 2017). Whether this population stands for mature monocytes with impaired function (deactivated monocytes) or for immature monocytes newly released into circulation, however, is still a matter of discussion and need to be further elucidated. Nevertheless, this pronounced alteration in the monocytic population in patients developing infections might lead to insufficient antigen presentation and costimulatory support for adaptive immune cells, overall decreasing the capacity to cope with infectious agents.

Mechanistically, both the autonomic nervous system and the HPA pathway have been proposed as the main triggers of these cell‐mediated loss of immunity. However, differences in their impact on poststroke infections have been encountered in experimental stroke. While both mechanisms contributed similarly to spleen atrophy, lymphocytopenia, and impaired monocytic function, only the inhibition of SNS, and not the blockage of the glucocorticoid receptors, minimize the occurrence of bacterial infections in ischemic mice after stroke (Prass *et al*, 2003). The attenuation of the cholinergic signaling pathway by either vagotomy or by using α7 nicotinic acetylcholine receptor‐deficient mice also reversed pulmonary immune low responsiveness and prevented poststroke pneumonia (Engel *et al*, 2015). Clinical studies could not provide evidence of a protective effect of the β‐blocker therapy: despite *Sykora et al* reported that prestroke and on‐stroke treatment with β‐blockers reduced the frequency of pneumonia after stroke (Sykora *et al*, 2015), others studies showed no differences in the development of post‐stroke pneumonia between patients with and without β‐blocker therapy (Maier *et al*, 2015, 2018; Westendorp *et al*, 2016). In view of these controversial findings, the causal relationship between the poststroke stress response, the subsequent immunosuppression, and the development of infections, which has been largely advocated within last decades, remains still questionable.

Other immune‐related mechanisms behind the stress response have also been evaluated in the context of poststroke infections. Recently, an inflammasome‐dependent mechanism of T cell apoptosis was found to have a crucial role in lymphopenia and the incidence of infections after stroke (Roth *et al*, 2021a). Specifically, the AIM2‐inflammasome activation in myeloid cells and the subsequent increase in IL‐1β levels early after stroke have been shown to promote the activation of monocytes and their expression of the FasL, which ultimately induce T cell apoptosis via a FasL–Fas‐mediated mechanism.

Therapeutically, a tight regulation of the stroke‐induced suppression of cellular immunity could be of clinical relevance for the prevention of post‐stroke complications. So far, the lack of success of the β‐blocker therapy urges the need for alternative therapies to tackle such life‐threatening post‐stroke comorbidities. In this regard, because DAMP and other pro‐inflammatory cytokines upregulated within the hyper‐acute inflammatory response to stroke are also considered to be key triggers of subacute immunosuppression, immunoregulatory approaches mitigating this early pro‐inflammatory reaction might ultimately be an alternative promising therapy to limit immunosuppression and poststroke infections. However, to date, no immunomodulation therapy has been clinically tested with the specific endpoint of reducing infections after stroke.

## Cardiovascular comorbidities and complications after stroke

Cardiovascular diseases, including atrial fibrillation, valvular heart disease, and congestive heart failure are well‐known risk factors for ischemic stroke (Chugh *et al*, 2014; Kim & Kim, 2018). However, this relationship is not unidirectional, since the rate of cardiovascular disorders also increases after first incident stroke (Kallmünzer *et al*, 2012; Buckley *et al*, 2022). Ischemic stroke patients are known to have an increased risk of vascular complications after their incident event, including vascular plaque formation and progression of atherosclerosis. Following stroke, more than 60% of patients also present electrocardiographic (ECG) abnormalities (Braga *et al*, 2020), 25% are detected with serious arrhythmia (Ruthirago *et al*, 2016), and about 19% of patients develop at least one serious cardiac adverse event (Prosser *et al*, 2007). Cardiac complications are not only frequent after ischemic stroke but also following other cerebrovascular events such as SAHs, and have been associated with worse clinical outcomes (Oras *et al*, 2016; Norberg *et al*, 2018; Buckley *et al*, 2022).

Inflammation is a shared key factor between stroke and cardiovascular disorders, and has been suggested to be closely involved in the development of cardiovascular comorbidities after an incident stroke (Willerson & Ridker, 2004). Key lines of evidence are provided by epidemiological studies showing a strong positive correlation between the levels of inflammatory markers and risk of cardiovascular events (Kaptoge *et al*, 2014). For instance, C‐C motif chemokine ligand 2/monocyte‐chemoattractant protein‐1 (CCL2/MCP1) is known to substantially increase after diverse cerebrovascular diseases including ischemic stroke, and its levels even positively correlate with detrimental patients' outcome (Geng *et al*, 2022). CCL2 has been proposed at the same time to play a key role in the development of diverse cardiovascular diseases, including the genesis and progression of atherosclerosis. In fact, large series of human studies have further provided evidence on the therapeutic potential of targeting the CCL2‐CCR2 (C‐C motif chemokine receptor 2) pathway in human atherosclerotic disease (Georgakis *et al*, 2022), which could also be relevant to minimize the risk of secondary cardiovascular complications after stroke.

Also, patients with chronic systemic inflammatory diseases, such as rheumatoid arthritis, psoriasis, or psoriatic arthritis, are known to develop an increased risk of insulin resistance, dyslipidemia, hypertension, and other cardiovascular events, including myocardial infarction, heart failure, or cerebrovascular injuries. In this line, a recent French nationwide population‐based cohort study also described a higher incidence of acute cardiovascular events in a cohort of 200,000 patients diagnosed with inflammatory bowel disease (Kirchgesner *et al*, 2018). In this study, cardiovascular risk was substantially increased even in the absence of the common well‐known cardiovascular risk factors, such as obesity, lipid disturbances, or hypertension, overall suggesting that inflammation could be a prominent shared key factor for the development of all these cardiovascular adverse manifestations. Hence, it is well conceivable that the systemic inflammatory response driven by the ischemic brain lesion itself could further predispose stroke patients for secondary (inflammatory) vascular events.

### Cardiovascular dysfunction

Current knowledge supports the hypothesis that there is a causal relationship between brain damage and cardiovascular dysfunction. Multiple mechanisms have been described to regulate this brain–heart interaction following stroke (Chen *et al*, 2017). The most acknowledged mechanisms are the stroke‐induced imbalance of the sympathetic and PNS. Sympathetic hyperactivity after stroke leads to a massive release of catecholamines, which directly activates β‐adrenergic receptors on myocardial nerves and provokes ectopic cardiac activity (Wang *et al*, 2019). Several clinical studies confirmed that this disturbance of the central autonomic pathway alters the physiological regulation and dynamics of the heart by decreasing heart rate variability (HRV), impairing baroreceptor reflex sensitivity (BRS) and further increasing the sympathetic and parasympathetic tone, with consequent cardiomyocyte toxicity (Chen *et al*, 2017). Despite the catecholamine hypothesis is the most widely proven mechanism of the brain–heart interaction so far, alternative mechanisms and particularly the immune system is emerging as a further critical factor playing a key role in the brain–heart communication after stroke.

Experimental animal studies have reported a higher incidence of cardiac dysfunction after ischemic stroke. Experimental stroke in mice resulted in chronic systolic dysfunction up to 8 weeks after the brain lesion, and caused a delayed reduction in left ventricular ejection fraction and an increase in left ventricular volume (Bieber *et al*, 2017; Veltkamp *et al*, 2019). Preclinical studies have also demonstrated a substantial increase in plasma catecholamine levels after cerebral ischemia. Molecularly, this disturbance of the catecholamine homeostasis has been shown to induce the upregulation of a distinct set of PPARγ‐dependent genes involved in mitochondrial remodeling, regulation of catabolism, and hypertrophy in the heart (Veltkamp *et al*, 2019). Other preclinical studies have also observed that cardiac damage after stroke might be a consequence of an impairment of the cardioprotective Survivor Activating Factor Enhancement (SAFE) signaling pathway (Meloux *et al*, 2018) and could also result from the disturbance of the calcium homeostasis in ventricular myocytes, which might ultimately restrict their contractile function (Sun *et al*, 2010).

The systemic immune response to stroke can be expected to have a direct impact on the heart and might also potentially cause cardiac dysfunction. The infiltration of pro‐inflammatory macrophages into the heart and the activation of the NLRP3 inflammasome pathway have been proposed as key events that could lead to cardiac dysfunction after ischemic stroke in mice (Lin *et al*, 2020). Interestingly, blocking the NLRP3 inflammasome in pro‐inflammatory macrophages restored cardiac function and reversed the myocardial morphological changes observed in mice after ischemic stroke (Lin *et al*, 2020). This close link between stroke‐induced systemic inflammation and heart dysfunction has been demonstrated to be even more pronounced in the presence of other comorbidities such as diabetes, which is likely to further promote the pro‐inflammatory immune response and the infiltration of immune cells to the heart due to enhanced vascular damage (Lin *et al*, 2020).

In addition, several pro‐inflammatory molecules upregulated after stroke have been also highlighted as plausible mediators of heart damage, based on their well‐known role in the heart after cardiac injury and other inflammatory diseases, including sepsis. This is the case for IL‐6, IL‐1β, TNF‐α, and IL‐18, whose negative inotropic effects on the heart have been extensively documented during last decades and previously reviewed in detail (Prabhu, 2004; Mann, 2015; Chen *et al*, 2017). Moreover, these pro‐inflammatory cytokines as well as other DAMP are also known to be able to activate cardiomyocytes through TLRs, which further increases the release of more pro‐inflammatory cytokines, sustain the inflammatory milieu, and could promote the development of cardiac dysfunction (Mann, 2015).

### Atherosclerosis

Atherosclerosis is an established cardiovascular risk factor predisposing patients to acute vascular events, including stroke (Parish *et al*, 2019). Similarly, patients who survive an incident stroke also have increased risk for recurrent vascular events, including recurrent strokes or myocardial infarctions, which are both common severe manifestations of atherosclerosis (Dhamoon *et al*, 2007). This bidirectional relationship between stroke and atherosclerosis indicates that beyond lipid disturbances, other mechanisms also promoted by stroke play a prominent role in the pathophysiology of atherosclerosis and vascular complications. In this regard, inflammation is known to play a critical role in the genesis, progression, and manifestation of atherosclerosis, as well as in the pathophysiology of ischemic stroke.

Numerous data support the role of inflammatory mechanisms in the formation of atherosclerosis across all disease stages (Geovanini & Libby, 2018). This pathophysiological process involves several steps, which includes: the activation of endothelial cells; the infiltration of monocytes to the atheroma; the secretion of pro‐inflammatory factors that further enhance the recruitment of immune cells; the formation of lipid‐rich macrophages and lipoprotein‐containing foam cells, which further secrete more pro‐inflammatory mediators; and the apoptosis of these macrophages and the formation of the mature lipid plaque, among others. Early findings in animal models of experimental myocardial infarction already documented that the systemic response to ischemic injury aggravates chronic atherosclerosis (Dutta *et al*, 2012). Disease progression was associated with a pronounced increased in monocyte recruitment, which was attributed to a massive egress of hematopoietic stem and progenitor cells from bone marrow niches mediated through sympathetic innervation (Dutta *et al*, 2012). More recently, exacerbation of atheroprogression has also been documented in the context of ischemic stroke (Roth *et al*, 2018). In a synergistic manner to the sympathetic stress response, the stroke‐induced release of DAMP from the ischemic brain has been found to be also critical for the exacerbation of vascular inflammation after stroke. While the sympathetic activation induced egress of monocytes from the bone marrow, circulating alarmins promoted the subsequent activation and infiltration of these monocytes into the vascular plaque (Roth *et al*, 2018). Recently, *Hettwer and colleagues* also reported an involvement of IL‐1β and the NLRP3‐inflammasome pathway in atheroprogression (Hettwer *et al*, 2021), which is a mechanism of likely relevance also for the inflammatory response after stroke. They demonstrated that IL‐1β and the NLRP3‐inflammasome pathway promote the expression of leukocyte chemoattractant factors and adhesion molecules on endothelial cells from atherosclerotic aortas, which substantially favors leukocyte infiltration. They also suggested that IL‐1β and the NLRP3‐inflammasome play a role in the proliferation of bone marrow hematopoietic stem and progenitor cells in the bone marrow, which increases the supply of inflammatory leukocytes to the blood. Indeed, anti‐IL‐1β treatment and NLRP3‐inflamasome inhibition in atherosclerotic mice reduced total plaque and necrotic core size, as well as the number of inflammatory leukocytes infiltrated in the atherosclerotic aortas (Hettwer *et al*, 2021).

NETs are also well‐known constituents of atherosclerotic lesions. Since the presence of circulating NETs released upon neutrophil activation following stroke is well documented (Vallés *et al*, 2017), poststroke NETs have been also proposed to be implicated in atherogenesis. To date, NETs are known to induce endothelial cell dysfunction, boost oxidative stress and the oxidation of high‐density lipoprotein particles, and promote immunothrombosis and the accumulation of prothrombotic molecules in the vessel wall, thus contributing to atheroma and thrombus formation (Moschonas & Tselepis, 2019; Stark & Massberg, 2021). Similarly, NETs in the atherosclerotic region might also prime macrophages for cytokine release, which further amplify immune cell recruitment in atherosclerotic plaques (Warnatsch *et al*, 2015). All these findings provide further evidence that after stroke, the pronounced increase in the levels of DAMPS, including NETs, circulating IL‐1β and the systemic activation of the inflammasome pathways might have a translationally relevant impact on atheroprogression and potential plaque destabilization.

Altogether, systemic inflammation is considered a crucial modifiable enhancer of atherosclerosis and the development of atherosclerotic plaques is now known to be potentially promoted by any medical condition or comorbidity that involve systemic inflammation, including the systemic response to stroke. Therefore, strategies pointing at the modulation of stroke‐induced chronic systemic alterations of the immune system might be of translational relevance for preventing the progression of atherosclerotic plaques and subsequent secondary vascular events.

### Diabetes

Patients with diabetes mellitus present more than double the risk of incident stroke (Luitse *et al*, 2012; Lau *et al*, 2019; Zabala *et al*, 2020). Also, 25 to 45% of ischemic stroke patients have diabetes mellitus at the time of first stroke, and stroke outcomes are worse among diabetic patients, resulting in increased mortality and morbidity (Dhamoon *et al*, 2007; Kernan *et al*, 2014; Lau *et al*, 2019).

Metabolic disturbances specifically contribute to post‐stroke complications adverse stroke outcomes. Hyperglycemia has been widely associated with brain infarct growth, edema formation, hemorrhagic transformation and less‐favorable neurological outcomes after ischemic stroke (Williams *et al*, 2002; Desilles *et al*, 2013; Broocks *et al*, 2019; Suissa *et al*, 2020). As the underlying mechanisms, data from preclinical experiments showed that hyperglycemia primes the thromboinflammatory cascade by activating the endothelium, platelets and neutrophils, and favors lactic acidosis and the accumulation of reactive oxygen species (ROS) (Zhang *et al*, 2016; Desilles *et al*, 2017). The consequent increase in microvascular thromboinflammation after stroke further contributes to alterations in cerebral blood flow and the permeability of the BBB, which can even provoke hemorrhagic transformations in the ischemic brain (Li *et al*, 2013).

Diabetic patients also show increased susceptibility to infections, including sepsis (Muller *et al*, 2005; Schuetz *et al*, 2011). Results from pre‐clinical studies suggest that diabetes impairs host defense by compromising bacterial clearance and the innate immune response by reducing adherence, chemotaxis and phagocytosis of circulating innate immune cells (Delamaire *et al*, 1997; Schuetz *et al*, 2011). Also, hyperglycemia and the consequent increase in glycation end products have been shown to contribute to abnormal pro‐inflammatory cytokine production and decreased T cell function, and compromise the expression of class I major histocompatibility complexes (MHC) on surface of myeloid cells, overall impairing cell immunity and the host's immune defense against secondary infections (Alves *et al*, 2012).

Diabetes also promotes atherogenesis. It is well established that hyperglycemia promotes glycation and oxidation of circulating lipoproteins and exacerbates vascular shear stress, which generally causes alterations in endothelial phenotype and contributes to endothelial dysfunction (Harja *et al*, 2008). Uncontrolled high glucose levels may also promote plaque growth and instability by enhancing ROS production and the activation of the NLRP3‐inflammasome signaling pathway, which further facilitates leukocyte infiltration to the vascular plaque (Sharma *et al*, 2018).

Yet from another perspective, chronic inflammation, and specifically immune cell activation, has been recognized as one of the contributing mechanisms to the development of insulin resistance and type‐2 diabetes (Tsalamandris *et al*, 2019). Based on this, it would be reasonable to speculate that the stroke‐induced low‐grade chronic inflammation in the late phase after stroke could also potentially alter the development of this metabolic comorbidity. Several lines of evidence support this concept. First, TNF‐α, IL‐6, IL‐1β, and IL‐18 are known to be key influential modulators of diabetes, as also cytokines which are upregulated during the systemic immune response to stroke. The specific link of this molecules to diabetes has been well‐described in obese humans presenting sustained inflammatory conditions. In such, macrophages within the adipose tissue produce these pro‐inflammatory cytokines that ultimately impair insulin signal and promote the progression of insulin resistance by downregulating the expression of glucose transporters and their translocation to the cell membrane in adipocytes (Zatterale *et al*, 2020) (Jager *et al*, 2007). Second, prolonged high levels of IL‐1β are known to also participate in insulin resistance by promoting pancreatic β‐cell dysfunction and cell death (Verma *et al*, 2013). Likewise, upstream of IL‐1β, the NLRP3 inflammasome pathway in myeloid cells also plays a part as a modulator of glucose metabolism and insulin resistance. In this regard, first pieces of evidence suggest that inhibition of NLRP3 inflammasome in obese mice improved both insulin signaling from adipose tissue and insulin secretion in the pancreas (Vandanmagsar *et al*, 2011; Wen *et al*, 2011) (Vandanmagsar *et al*, 2011; Tsalamandris *et al*, 2019). Based on these findings, future research should elucidate whether the stroke‐induced signaling through IL‐1β and NLRP3 inflammasome pathway could potentially trigger and exacerbate disturbances in the glucose metabolism in ischemic stroke patients.

Altogether, these concepts reviewed above reinforce the close connection between chronic inflammation from diverse etiologies, including stroke and metabolic disturbances. Specifically, co‐occurrence of diabetes and ischemic stroke are now known to potentially activate diverse inflammatory pathways that share common immune mediators, ultimately boosting the inflammatory reaction to stroke and leading to a higher susceptibility to other inflammation‐associated comorbidities.

## Targeting systemic immunity to improve post‐stroke complications and comorbidities

Stroke promotes systemic inflammation, and growing evidence indicates that systemic inflammation represents a risk factor for the development of other inflammation‐related diseases, including cardiovascular events. Thus, it is plausible that some stroke comorbidities could partly either arise as a result of or be further exacerbated by the systemic immune response originated after an initial stroke event. Indeed, accumulating evidence suggest that conserved immunological mechanisms might play similar roles in different diseases of diverse nature that encompass systemic inflammation, further supporting a plausible effect of the stroke‐induced immunological response on the progression of other inflammation‐associated diseases (Hoyer *et al*, 2019; Roth *et al*, 2021a). Under this rationale, targeting post‐stroke chronic inflammation has currently emerged as a promising therapeutic intervention to ultimately improve stroke outcome, prevent recurrent vascular events and avoid secondary comorbidities (Table 1).

**Table 1:** Clinical trials targeting systemic inflammation in patients with ischemic stroke.

Drug	MoA	Trial acronym	Unique identifier	Patients characteristics	PMID	Primary outcome	Observed outcome
ApTOLL	TLR4 antagonist		NCT04734548	Acute ischemic stroke patients treated with EV therapy	–	Adverse events at d90 (death, recurrent stroke, sICH)	Ongoing
Colchicine	Microtubule polymerization inhibitor	CONVINCE	NCT02898610	Non‐cardioembolic ischemic stroke or high‐risk TIA patients	34414298	Recurrent non‐fatal and fatal stroke or MI within 60 months	Ongoing
Dimethyl fumarate	Multi‐target anti‐oxidant and immunomodulatory mechanisms		NCT04890366	Acute ischemic stroke patients treated with alteplase	–	Changes in lesion volume, HT and neurological impairment at d1 (NHISS)	Ongoing
			NCT04891497	Acute ischemic stroke patients treated with EV therapy	–	Changes in lesion volume, HT and neurological impairment at d1 (NIHSS)	Ongoing
Enlimomab	Anti‐ICAM‐1 antibody			Acute ischemic stroke patients	11673584		Worse outcome (d5, d30, d90)
Anakinra	IL‐1R antagonist	SCIL‐Stroke	ISRCTN74236229	Acute ischemic stroke patients	29567761	Concentration of plasma IL‐6 at d3	Reduced plasma inflammatory markers (IL‐6 and CRP)
Indobufen	platelet aggregation inhibitor	INSURE	NCT03871517	Ischemic stroke patients	35393360	Recurrent stroke and moderate bleeding at 90d	Ongoing
Natalizumab	Anti‐VLA‐4 antibody	ACTION	NCT01955707	Acute ischemic stroke patients	28229893	Changes in lesion volume (baseline vs. d5)	No reduction of infarct volume (d5, d30)
		ACTION2	NCT02730455	Acute ischemic stroke patients	32591475	Favorable outcome at d90 (mRS)	No outcome improvement (d90)
Pioglitazone	PPAR synthetic ligand	IRIS	NCT00091949	Ischemic stroke or TIA patients	25458644 29084736	Recurrent non‐fatal and fatal stroke or MI at 5 years	Reduced risk of secondary ischemic stroke (5 years)
			NCT04123067	Hyperglycemic acute ischemic stroke patients	–	Clinical improvement at d90 (NIHSS, mRS)	Ongoing
Rivaroxaban	Inhibits coagulation factor Xa	ESUS	NCT02313909	Ischemic stroke patients	29766772	Incident events (stroke, TIA, embolism)	Terminated due to no efficacy
		RISAPS	NCT03684564	Ischemic stroke patients with persistent antiphospholipid antibodies	–	Change in brain WMH volume at 24 months	Ongoing
Vipocentine	Inhibitor of Calcium‐dependent cyclic‐GMP metabolism		NCT02878772	Ischemic stroke patients	28691141	Changes in lesion volume (baseline vs. d7), brain inflammatory level, clinical improvement at d7 and d14 (NIHSS)	Reduced secondary lesion enlargement, attenuated neuroinflammation, improved outcome (3 m)

Abbreviations: EV, endovascular; GCS, Glasgow coma scale; GMP, guanosine 3′,5′‐cyclic monophosphate; HT, hemorrhagic transformation; ICAM‐1, intracellular adhesion molecule‐1; IL‐1R, Interleukin‐1 receptor; MAG, myelin‐associated glycoprotein; MI, myocardial infarction; mBI, modified Barthel Index; mRS, modified rankin scale; NHISS, National Institute of Health stroke scale/score; PPAR, peroxisome proliferator‐activated receptors; sIHC, symptomatic intracranial hemorrhage; S1P, sphingosine‐1‐phosphate; TIA, transient ischemic attach; TLR4, Toll‐like receptor‐4; VLA‐4, integrin α4β1; WMH, white matter hyperintensity.

Several pro‐inflammatory mediators such as TNF‐α, IL‐1β, and IL‐6 have been proposed as key drivers of secondary vascular events after stroke. Since very limited data is available from stroke trials so far, a first line of evidence proving the potential beneficial effect of targeting these pro‐inflammatory molecules on common poststroke comorbidities has been inferred from clinical trials for other diseases that primarily manifest a chronic inflammatory response with shared immunological mechanisms. For instance, the use of TNF‐α inhibitors in patients with psoriasis and psoriatic arthritis has been shown to provide net benefits with regard to the risk of developing cardiovascular events (Yang *et al*, 2016). Similarly, the incidence of other stroke‐associated comorbidities such as diabetes mellitus and dementia is known to be reduced by anti‐TNF‐α and anti‐IL‐1β therapy in patients with rheumatoid arthritis and comorbid type‐2 diabetes (Chou *et al*, 2016) (Burska *et al*, 2015). Analogously to these diseases, targeting systemic immunity after stroke seems thus a promising strategy to attenuate the incidence of poststroke secondary comorbidities, despite the degree and extent of the inflammatory response across all these different diseases is still uncertain and may vary from one pathology to another.

A second and more solid line of evidence comes from several randomized controlled clinical trials that have directly evaluated systemic immunity as a key target in patients suffering from diverse incident vascular diseases. For instance, several proof‐of‐concept clinical studies have assessed the effects of the pharmacological blockage of IL‐1β signaling pathway via the administration of Anakinra, a recombinant human interleukin‐1–receptor antagonist (IL‐1Ra), in diverse cerebrovascular diseases. The recent SCIL‐STROKE and SCIL‐SAH trials (Subcutaneous Interleukin‐1 Receptor Antagonist in Ischemic Stroke and aneurysmal SAH, respectively) two small single‐center double‐blind randomized phase 2 trials, demonstrated the efficacy of subcutaneous IL‐1Ra administration in reducing the peripheral inflammatory response, evaluated by plasma IL‐6 and CRP levels, in acute ischemic stroke and SAH, respectively (Galea *et al*, 2018; Smith *et al*, 2018). In this same line, another clinical trial evaluating the effect of IL‐1Ra on lowering inflammation after spontaneous intracerebral hemorrhage is also currently ongoing (Studying Anakinra to Reduce Secondary Brain Damage After Spontaneous Hemorrhagic Stroke (ACTION, NCT04834388)). Beyond these cerebrovascular diseases, Anakinra has been also tested in clinical trials for other acute brain diseases like severe TBI. In such, a detailed panel of cytokines and chemokines were evaluated in cerebral microdialysates and arterial and jugular venous samples from TBI patients treated with subcutaneous IL‐1Ra or placebo (Helmy *et al*, 2014; Lassarén *et al*, 2021). IL‐1Ra treatment resulted in a notable attenuation of the neuroinflammatory response in brain, although far fewer differences were observed in the cytokine response to TBI at the systemic level for the treatment group (Lassarén *et al*, 2021). Furthermore, beyond brain diseases, Anakinra has proved to be effective in reducing inflammation in patients with rheumatoid arthritis and type‐2 diabetes (Ruscitti *et al*, 2019).

The CANTOS trial (Canakinumab Anti‐inflammatory Thrombosis Outcome Study), a multinational double‐blind phase III study, was also designed to target inflammation in patients with established atherosclerotic disease who had prior myocardial infarction and high residual systemic inflammatory state (measured by high‐sensitivity CRP levels >2 mg/L)—comparable to the chronic inflammatory state after stroke (Ridker *et al*, 2017). Particularly, the CANTOS trial tested efficacy of reducing inflammation by neutralizing IL‐1β with Canakinumab, a full human monoclonal antibody. Canakinumab significantly reduced the incidence of cardiovascular events, including myocardial infarction, stroke and cardiovascular death. These beneficial effects were directly attributed to a lower systemic inflammatory response, since treatment with canakinumab did not show any effect on other well‐known cardiovascular risk factors such as lipids or blood pressure levels. Although results from the CANTOS study supported the notion that reducing vascular inflammation in the absence of concomitant lipid lowering reduces the rates of recurrent vascular events, the mechanisms underlying these beneficial effects remain incompletely understood. Importantly, IL‐1β neutralization in CANTOS significantly increased the frequency of global fatal infections and sepsis. These findings would argue towards a cautious use and further translation of such immunomodulatory approaches for use in patients with increased susceptibility to infections, such as stroke patients; therefore, more specific approaches that block specific pathways leading to subacute immune exhaustion rather than also inhibiting potential effector functions such as IL‐1β might be more promising for future development in stroke patients.

Beyond IL‐1β, other anti‐inflammatory strategies are also now under investigation to lower inflammation and prevent cardiovascular events. This is the case of colchicine, a widely available, safe, and low‐cost anti‐inflammatory drug currently used as a treatment for gout, Behçet's disease and familiar Mediterranean fever. Mechanistically, colchicine is known to inhibit tubulin polymerization and microtubule generation, which primarily impairs cell mitosis and motility. Moreover, colchicine has also been known to interfere with the inflammatory process by inhibiting the synthesis of pro‐inflammatory factors, including TNF‐α, COX‐2, and E‐ and P‐selectin expression, among others, and impairing the activity of the NLRP3 inflammasome by blocking its oligomerization (Martínez *et al*, 2018). These latest mechanisms of action of colchicine pointed to the fact that it might also potentially be used to reduce residual inflammation in cardiovascular diseases. In this regard, several clinical trials demonstrated that colchicine treatment is safe and effective for the prevention of secondary vascular events in patients with stable coronary disease or myocardial infarction (Nidorf *et al*, 2013; Tardif *et al*, 2019). Recent meta‐analyses performed on all available data from previous randomized controlled trials also demonstrated that colchicine treatment results in a significant reduction of stroke incidence in patients with high cardiovascular risk (Katsanos *et al*, 2020; Masson *et al*, 2020). Based on these first evidences in the cardiovascular field, the effect of this anti‐inflammatory treatment on the prevention of secondary adverse events following stroke is currently under investigation. An international multicenter, prospective, randomized phase 3 clinical trial (CONVINCE, Colchicine for Prevention of Vascular Inflammation in Non‐cardio Embolic Stroke) testing the efficacy of colchicine in ischemic stroke patients with an anticipated median follow up of 36 month is currently ongoing (Kelly *et al*, 2021). The primary outcome of this clinical study is time to first recurrent nonfatal ischemic stroke, nonfatal cardiac events or fatal cardiovascular deaths. Similarly, colchicine is also currently being evaluated as a potential therapeutic intervention to reduce residual vascular risk in patients with peripheral arterial disease in another double‐blind randomized phase 3 clinical trial (LEADER‐PAD, Low Dose ColchicinE in pAtients with Peripheral Arterial Disease to Address Residual Vascular Risk).

Taken together, the existing first clinical data from studies targeting systemic inflammation in multiple inflammation‐related diseases suggest that systemic inflammation is as an important driver of secondary vascular events in patients with underlying vascular comorbidities and preceding ischemic events. The currently ongoing and newly planned studies aiming at preventing vascular events after stroke by means of anti‐inflammatory strategies further highlight that the relevance of systemic inflammation after stroke has been already recognized by the large stroke research field, and will likely motivate further trials to test alternative anti‐inflammatory approaches and drugs for the prevention of post‐stroke comorbidities.

## Conclusions

In recent years, systemic immunity has gained significant importance as a key player in the stroke pathology. Despite its increasing relevance, no consideration is yet given to systemic inflammation for the management of stroke patients in the clinical practice. Solid evidence points toward a tight connection between poststroke systemic inflammation and secondary complications, including infections and diverse cardiovascular pathologies. This emerging concept suggests that inflammation could be a potential target for intervention in stroke patients to improve stroke outcomes and avoid secondary complications at the same time. Particularly, targeting IL‐1β has shown promising results so far. The CANTOS study laid the foundation for the prevention of cardiovascular comorbidities by minimizing systemic inflammation and showed a clear benefit for patients with a high‐risk cardiovascular profile. Similarly, the beneficial effects of Colchicine for preventing secondary vascular events also support the promising potential of anti‐inflammatory agents as therapeutic approaches to minimize the incidence of cerebrovascular and cardiovascular diseases in patients of high risk. Several clinical trials for anti‐inflammatory strategies in stroke are currently recruiting and we can expect increasing interest in the systemic immune response as a therapeutic target. This development gives hope for a paradigm shift from a neuro‐centric view on stroke therapy toward the recognition of systemic effects as important elements in the personalized treatment of stroke patients.

## Author contributions

**Alba Simats:** Conceptualization; writing – original draft; writing – review and editing. **Arthur Liesz:** Conceptualization; writing – original draft; writing – review and editing.

In addition to the CRediT author contributions listed above, the contributions in detail are:

AS and AL contributed to the conception, writing, and editing of the manuscript.

## Disclosure and competing interests statement

The authors declare that they have no conflict of interest.

**For more information**

i American Heart Association is a voluntary organization dedicated to fighting heart disease and stroke—www.heart.org
ii ClinicalTrials.gov is a database of privately and publicly funded clinical studies conducted around the world—www.clinicaltrials.gov
iii ImmunoStroke consortium: DFG‐funded consortium for analyzing brain‐immune interaction in stroke—https://immunostroke.de

**Pending issues**

- Uncovering the source of increased systemic pro‐inflammatory mediators across time after ischemic stroke and their association with stroke severity and etiology.
- Understanding in detail the impact of each specific chronic pro‐inflammatory mediator on the pre‐existing or acquired comorbidities after ischemic stroke.
- Understanding the interaction between primary immunological mechanisms after stroke with other pathways such as stress response or neurohumoral signals in modulating post‐stroke immunity.
- Elucidating the effect of targeting systemic inflammation as a therapeutic approach to minimize the incidence of secondary comorbidities chronically after stroke.

## Supporting information



Table EV1

## References

[emmm202216269-bib-0001] Abdullahi W , Tripathi D , Ronaldson PT (2018) Blood‐brain barrier dysfunction in ischemic stroke: targeting tight junctions and transporters for vascular protection. Am J Physiol Cell Physiol 315: C343–C356 29949404 10.1152/ajpcell.00095.2018 PMC6171039

[emmm202216269-bib-0002] Ajmo CT , Collier LA , Leonardo CC , Hall AA , Green SM , Womble TA , Cuevas J , Willing AE , Pennypacker KR (2009) Blockade of adrenoreceptors inhibits the splenic response to stroke. Exp Neurol 218: 47–55 19371742 10.1016/j.expneurol.2009.03.044 PMC2720830

[emmm202216269-bib-0003] Alishahi M , Farzaneh M , Ghaedrahmati F , Nejabatdoust A , Sarkaki A , Khoshnam SE (2019) NLRP3 inflammasome in ischemic stroke: as possible therapeutic target. Int J Stroke 14: 574–591 30940045 10.1177/1747493019841242

[emmm202216269-bib-0004] Alves C , Casqueiro J , Casqueiro J (2012) Infections in patients with diabetes mellitus: a review of pathogenesis. Indian J Endocrinol Metab 16: 27–S36 10.4103/2230-8210.94253 PMC3354930 22701840

[emmm202216269-bib-0005] Andersson U , Tracey KJ (2011) HMGB1 is a therapeutic target for sterile inflammation and infection. Annu Rev Immunol 29: 139–162 21219181 10.1146/annurev-immunol-030409-101323 PMC4536551

[emmm202216269-bib-0006] Anrather J , Iadecola C (2016) Inflammation and stroke: an overview. Neurotherapeutics 13: 661–670 27730544 10.1007/s13311-016-0483-x PMC5081118

[emmm202216269-bib-0007] Aref HMA , Fahmy NA , Khalil SH , Ahmed MF , ElSadek A , Abdulghani MO (2020) Role of interleukin‐6 in ischemic stroke outcome. Egypt J Neurol Psychiatr Neurosurg 56: 12

[emmm202216269-bib-0008] Ashour W , Al‐Anwar AD , Kamel AE , Aidaros MA (2016) Predictors of early infection in cerebral ischemic stroke. J Med Life 9: 163–169 27453748 PMC4863508

[emmm202216269-bib-0009] Basic Kes V , Simundic AM , Nikolac N , Topic E , Demarin V (2008) Pro‐inflammatory and anti‐inflammatory cytokines in acute ischemic stroke and their relation to early neurological deficit and stroke outcome. Clin Biochem 41: 1330–1334 18801351 10.1016/j.clinbiochem.2008.08.080

[emmm202216269-bib-0010] Benakis C , Brea D , Caballero S , Faraco G , Moore J , Murphy M , Sita G , Racchumi G , Ling L , Pamer EG *et al* (2016) Commensal microbiota affects ischemic stroke outcome by regulating intestinal γδ T cells. Nat Med 22: 516–523 27019327 10.1038/nm.4068 PMC4860105

[emmm202216269-bib-0011] Benakis C , Poon C , Lane D , Brea D , Sita G , Moore J , Murphy M , Racchumi G , Iadecola C , Anrather J (2020) Distinct commensal bacterial signature in the gut is associated with acute and long‐term protection from ischemic stroke. Stroke 51: 1844–1854 32404038 10.1161/STROKEAHA.120.029262 PMC7810258

[emmm202216269-bib-0012] Bieber M , Werner RA , Tanai E , Hofmann U , Higuchi T , Schuh K , Heuschmann PU , Frantz S , Ritter O , Kraft P *et al* (2017) Stroke‐induced chronic systolic dysfunction driven by sympathetic overactivity. Ann Neurol 82: 729–743 29023958 10.1002/ana.25073 PMC5765487

[emmm202216269-bib-0013] Borovikova LV , Ivanova S , Zhang M , Yang H , Botchkina GI , Watkins LR , Wang H , Abumrad N , Eaton JW , Tracey KJ (2000) Vagus nerve stimulation attenuates the systemic inflammatory response to endotoxin. Nature 405: 458–462 10839541 10.1038/35013070

[emmm202216269-bib-0014] Bower NI , Hogan BM (2018) Brain drains: new insights into brain clearance pathways from lymphatic biology Drainage and clearance pathways of the central nervous system. J Mol Med 96: 383–390 29610928 10.1007/s00109-018-1634-9

[emmm202216269-bib-0015] Braga GP , Gonçalves RS , Minicucci MF , Bazan R , Zornoff LAM (2020) Strain pattern and T‐wave alterations are predictors of mortality and poor neurologic outcome following stroke. Clin Cardiol 43: 568–573 32087617 10.1002/clc.23348 PMC7298998

[emmm202216269-bib-0016] Brea D , Poon C , Benakis C , Lubitz G , Murphy M , Iadecola C , Anrather J (2021) Stroke affects intestinal immune cell trafficking to the central nervous system. Brain Behav Immun 96: 295–302 33989742 10.1016/j.bbi.2021.05.008 PMC8672365

[emmm202216269-bib-0017] Broocks G , Kemmling A , Aberle J , Kniep H , Bechstein M , Flottmann F , Leischner H , Faizy TD , Nawabi J , Schön G *et al* (2019) Ischemic lesion water uptake in acute stroke: is blood glucose related to cause and effect? J Stroke 21: 347–349 31590479 10.5853/jos.2019.01935 PMC6780013

[emmm202216269-bib-0018] Buckley BJR , Harrison SL , Hill A , Underhill P , Lane DA , Lip GYH (2022) Stroke‐heart syndrome: incidence and clinical outcomes of cardiac complications following stroke. Stroke 53: 1759–1763 35354300 10.1161/STROKEAHA.121.037316

[emmm202216269-bib-0019] Burska AN , Sakthiswary R , Sattar N (2015) Effects of tumour necrosis factor antagonists on insulin sensitivity/resistance in rheumatoid arthritis: a systematic review and meta‐analysis. PLoS One 10: 1–10 10.1371/journal.pone.0128889 PMC4482317 26110878

[emmm202216269-bib-0020] Bustamante A , Sobrino TT , Giralt D , García‐Berrocoso T , Llombart V , Ugarriza I , Espadaler M , Rodríguez N , Sudlow C , Castellanos M *et al* (2014) Prognostic value of blood interleukin‐6 in the prediction of functional outcome after stroke: a systematic review and meta‐analysis. J Neuroimmunol 274: 215–224 25091431 10.1016/j.jneuroim.2014.07.015

[emmm202216269-bib-0021] Bustamante A , Giralt D , García‐Berrocoso T , Rubiera M , Álvarez‐Sabín J , Molina C , Serena J , Montaner J (2017) The impact of post‐stroke complications on in‐hospital mortality depends on stroke severity. Eur Stroke J 2: 54–63 31008302 10.1177/2396987316681872 PMC6453178

[emmm202216269-bib-0022] Chaban V , Clarke GJB , Skandsen T , Islam R , Einarsen CE , Vik A , Damås JK , Mollnes TE , Håberg AK , Pischke SE (2020) Systemic inflammation persists the first year after mild traumatic brain injury: results from the prospective trondheim mild traumatic brain injury study. J Neurotrauma 37: 2120–2130 32326805 10.1089/neu.2019.6963 PMC7502683

[emmm202216269-bib-0023] Chamorro Á , Amaro S , Vargas M , Obach V , Cervera Á , Torres F , Planas AM (2006) Interleukin 10, monocytes and increased risk of early infection in ischaemic stroke. J Neurol Neurosurg Psychiatry 77: 1279–1281 17043295 10.1136/jnnp.2006.100800 PMC2077369

[emmm202216269-bib-0024] Chen Z , Venkat P , Seyfried D , Chopp M , Yan T , Chen J (2017) Brain‐heart interaction: cardiac complications after stroke. Circ Res 121: 451–468 28775014 10.1161/CIRCRESAHA.117.311170 PMC5553569

[emmm202216269-bib-0025] Chiu NL , Kaiser B , Nguyen YV , Welbourne S , Lall C , Cramer SC (2016) The volume of the spleen and its correlates after acute stroke. J Stroke Cerebrovasc Dis 25: 2958–2961 27615448 10.1016/j.jstrokecerebrovasdis.2016.08.012 PMC5154801

[emmm202216269-bib-0026] Chou RC , Kane M , Ghimire S , Gautam S , Gui J (2016) Treatment for rheumatoid arthritis and risk of Alzheimer's disease: a nested case‐control analysis. CNS Drugs 30: 1111–1120 27470609 10.1007/s40263-016-0374-z PMC5585782

[emmm202216269-bib-0027] Chugh SS , Havmoeller R , Narayanan K , Singh D , Rienstra M , Benjamin EJ , Gillum RF , Kim YH , McAnulty JH , Zheng ZJ *et al* (2014) Worldwide epidemiology of atrial fibrillation: a global burden of disease 2010 study. Circulation 129: 837–847 24345399 10.1161/CIRCULATIONAHA.113.005119 PMC4151302

[emmm202216269-bib-0028] Courties G , Herisson F , Sager HB , Heidt T , Ye Y , Wei Y , Sun Y , Severe N , Dutta P , Scharff J *et al* (2015) Ischemic stroke activates hematopoietic bone marrow stem cells. Circ Res 116: 407–417 25362208 10.1161/CIRCRESAHA.116.305207 PMC4312511

[emmm202216269-bib-0029] Courties G , Frodermann V , Honold L , Zheng Y , Herisson F , Schloss MJ , Sun Y , Presumey J , Severe N , Engblom C *et al* (2019) Glucocorticoids regulate bone marrow B lymphopoiesis after stroke. Circ Res 124: 1372–1385 30782088 10.1161/CIRCRESAHA.118.314518 PMC6483874

[emmm202216269-bib-0030] De Meyer SF , Denorme F , Langhauser F , Geuss E , Fluri F , Kleinschnitz C (2016) Thromboinflammation in stroke brain damage. Stroke 47: 1165–1172 26786115 10.1161/STROKEAHA.115.011238

[emmm202216269-bib-0031] Delamaire M , Maugendre D , Moreno M , Le Goff MC , Allannic H , Genetet B (1997) Impaired leucocyte functions in diabetic patients. Diabet Med 14: 29–34 9017350 10.1002/(SICI)1096-9136(199701)14:1<29::AID-DIA300>3.0.CO;2-V

[emmm202216269-bib-0032] Denorme F , Portier I , Rustad JL , Cody MJ , de Araujo CV , Hoki C , Alexander MD , Grandhi R , Dyer MR , Neal MD *et al* (2022) Neutrophil extracellular traps regulate ischemic stroke brain injury. J Clin Investig 132 10.1172/JCI154225 PMC9106355 35358095

[emmm202216269-bib-0033] Desilles J‐P , Meseguer E , Labreuche J , Lapergue B , Sirimarco G , Gonzalez‐Valcarcel J , Lavallée P , Cabrejo L , Guidoux C , Klein I *et al* (2013) Diabetes mellitus, admission glucose, and outcomes after stroke thrombolysis. Stroke 44: 1915–1923 23704108 10.1161/STROKEAHA.111.000813

[emmm202216269-bib-0034] Desilles JP , Syvannarath V , Ollivier V , Journé C , Delbosc S , Ducroux C , Boisseau W , Louedec L , Di Meglio L , Loyau S *et al* (2017) Exacerbation of thromboinflammation by hyperglycemia precipitates cerebral infarct growth and hemorrhagic transformation. Stroke 48: 1932–1940 28526762 10.1161/STROKEAHA.117.017080

[emmm202216269-bib-0035] Dhamoon MS , Tai W , Boden‐Albala B , Rundek T , Paik MC , Sacco RL , Elkind MSV (2007) Risk of myocardial infarction or vascular death after first ischemic stroke: the northern Manhattan study. Stroke 38: 1752–1758 17431206 10.1161/STROKEAHA.106.480988

[emmm202216269-bib-0036] Dutta P , Courties G , Wei Y , Leuschner F , Gorbatov R , Robbins CS , Iwamoto Y , Thompson B , Carlson AL , Heidt T *et al* (2012) Myocardial infarction accelerates atherosclerosis. Nature 487: 325–329 22763456 10.1038/nature11260 PMC3401326

[emmm202216269-bib-0037] Dziedzic T (2015) Systemic inflammation as a therapeutic target in acute ischemic stroke. Expert Rev Neurother 15: 523–531 25865856 10.1586/14737175.2015.1035712

[emmm202216269-bib-0038] Engel O , Akyüz L , Da Costa Goncalves AC , Winek K , Dames C , Thielke M , Herold S , Böttcher C , Priller J , Volk HD *et al* (2015) Cholinergic pathway suppresses pulmonary innate immunity facilitating pneumonia after stroke. Stroke 46: 3232–3240 26451017 10.1161/STROKEAHA.115.008989

[emmm202216269-bib-0039] Engelmann B , Massberg S (2013) Thrombosis as an intravascular effector of innate immunity. Nat Rev Immunol 13: 34–45 23222502 10.1038/nri3345

[emmm202216269-bib-0040] Esposito E , Ahn BJ , Shi J , Nakamura Y , Park JH , Mandeville ET , Yu Z , Chan SJ , Desai R , Hayakawa A *et al* (2019) Brain‐to‐cervical lymph node signaling after stroke. Nat Commun 10: 5306 31757960 10.1038/s41467-019-13324-w PMC6876639

[emmm202216269-bib-0041] Faura J , Bustamante A , Miró‐Mur F , Montaner J (2021) Stroke‐induced immunosuppression: implications for the prevention and prediction of post‐stroke infections. J Neuroinflammation 18: 1–14 34092245 10.1186/s12974-021-02177-0 PMC8183083

[emmm202216269-bib-0042] Feigin VL , Stark BA , Johnson CO , Roth GA , Bisignano C , Abady GG , Abbasifard M , Abbasi‐Kangevari M , Abd‐Allah F , Abedi V *et al* (2021) Global, regional, and national burden of stroke and its risk factors, 1990–2019: a systematic analysis for the Global Burden of Disease Study 2019. Lancet Neurol 20: 795–820 34487721 10.1016/S1474-4422(21)00252-0 PMC8443449

[emmm202216269-bib-0043] Galea J , Ogungbenro K , Hulme S , Patel H , Scarth S , Hoadley M , Illingworth K , McMahon CJ , Tzerakis N , King AT *et al* (2018) Reduction of inflammation after administration of interleukin‐1 receptor antagonist following aneurysmal subarachnoid hemorrhage: results of the Subcutaneous Interleukin‐1Ra in SAH (SCIL‐SAH) study. J Neurosurg 128: 515–523 28298024 10.3171/2016.9.JNS16615

[emmm202216269-bib-0044] Gallacher KI , Batty GD , McLean G , Mercer SW , Guthrie B , May CR , Langhorne P , Mair FS (2014) Stroke, multimorbidity and polypharmacy in a nationally representative sample of 1,424,378 patients in Scotland: implications for treatment burden. BMC Med 12: 1–9 10.1186/s12916-014-0151-0 PMC4220053 25280748

[emmm202216269-bib-0045] Gallacher KI , Jani BD , Hanlon P , Nicholl BI , Mair FS (2019) Multimorbidity in stroke. Stroke 50: 1919–1926 31233391 10.1161/STROKEAHA.118.020376

[emmm202216269-bib-0046] Garcia‐Bonilla L , Moore JM , Racchumi G , Zhou P , Butler JM , Iadecola C , Anrather J (2014) Inducible nitric oxide synthase in neutrophils and endothelium contributes to ischemic brain injury in mice. J Immunol 193: 2531–2537 25038255 10.4049/jimmunol.1400918 PMC4147670

[emmm202216269-bib-0047] Garlichs CD , Kozina S , Fateh‐Moghadam S , Tomandl B , Stumpf C , Eskafi S , Raaz D , Schmeißer A , Yilmaz A , Ludwig J *et al* (2003) Upregulation of CD40‐CD40 ligand (CD154) in patients with acute cerebral ischemia. Stroke 34: 1412–1417 12764232 10.1161/01.STR.0000074032.64049.47

[emmm202216269-bib-0048] Geng H , Chen L , Tang J , Chen Y , Wang L (2022) The role of CCL2/CCR2 axis in cerebral ischemia‐reperfusion injury and treatment: from animal experiments to clinical trials. Int J Mol Sci 23: 3485 35408846 10.3390/ijms23073485 PMC8998625

[emmm202216269-bib-0049] Georgakis MK , Bernhagen J , Heitman LH , Weber C , Dichgans M (2022) Targeting the CCL2–CCR2 axis for atheroprotection. Eur Heart J 43: 1799–1808 35567558 10.1093/eurheartj/ehac094

[emmm202216269-bib-0050] Geovanini GR , Libby P (2018) Atherosclerosis and inflammation: overview and updates. Clin Sci 132: 1243–1252 10.1042/CS20180306 29930142

[emmm202216269-bib-0051] Gruden G , Barutta F , Catto I , Bosco G , Caprioli MG , Pinach S , Fornengo P , Cavallo‐Perin P , Davini O , Cerrato P *et al* (2013) Serum levels of heat shock protein 27 in patients with acute ischemic stroke. Cell Stress Chaperones 18: 531–533 23334892 10.1007/s12192-013-0403-5 PMC3682014

[emmm202216269-bib-0052] Haeusler KG , Schmidt WUH , Föhring F , Meisel C , Helms T , Jungehulsing GJ , Nolte CH , Schmolke K , Wegner B , Meisel A *et al* (2008) Cellular immunodepression preceding infectious complications after acute ischemic stroke in humans. Cerebrovasc Dis 25: 50–58 18033958 10.1159/000111499

[emmm202216269-bib-0053] Harja E , Bu DX , Hudson BI , Jong SC , Shen X , Hallam K , Kalea AZ , Lu Y , Rosario RH , Oruganti S *et al* (2008) Vascular and inflammatory stresses mediate atherosclerosis via RAGE and its ligands in apoE^−/−^ mice. J Clin Investig 118: 183–194 18079965 10.1172/JCI32703 PMC2129235

[emmm202216269-bib-0054] Helmy A , Guilfoyle MR , Carpenter KLH , Pickard JD , Menon DK , Hutchinson PJ (2014) Recombinant human interleukin‐1 receptor antagonist in severe traumatic brain injury: a phase II randomized control trial. J Cereb Blood Flow Metab 34: 845–851 24569690 10.1038/jcbfm.2014.23 PMC4013762

[emmm202216269-bib-0055] Hettwer J , Hinterdobler J , Miritsch B , Deutsch M‐A , Li X , Mauersberger C , Moggio A , Braster Q , Gram H , Robertson AAB *et al* (2021) Interleukin‐1β suppression dampens inflammatory leukocyte production and uptake in atherosclerosis. Cardiovasc Res 1: 105–112 10.1093/cvr/cvab337 PMC9586563 34718444

[emmm202216269-bib-0056] Hoffmann S , Harms H , Ulm L , Nabavi DG , Mackert B‐M , Schmehl I , Jungehulsing GJ , Montaner J , Bustamante A , Hermans M *et al* (2017) Stroke‐induced immunodepression and dysphagia independently predict stroke‐associated pneumonia – The PREDICT study. J Cereb Blood Flow Metab 37: 3671–3682 27733675 10.1177/0271678X16671964 PMC5718319

[emmm202216269-bib-0057] Holmegaard L , Stanne TM , Andreasson U , Zetterberg H , Blennow K , Blomstrand C , Jood K , Jern C (2021) Proinflammatory protein signatures in cryptogenic and large artery atherosclerosis stroke. Acta Neurol Scand 143: 303–312 33107019 10.1111/ane.13366 PMC7898473

[emmm202216269-bib-0058] Honarpisheh P , Bryan RM , McCullough LD (2022) Aging microbiota‐gut‐brain axis in stroke risk and outcome. Circ Res 130: 1112–1144 35420913 10.1161/CIRCRESAHA.122.319983 PMC9674376

[emmm202216269-bib-0059] Houlden A , Goldrick M , Brough D , Vizi ES , Lénárt N , Martinecz B , Roberts IS , Denes A (2016) Brain injury induces specific changes in the caecal microbiota of mice via altered autonomic activity and mucoprotein production. Brain Behav Immun 57: 10–20 27060191 10.1016/j.bbi.2016.04.003 PMC5021180

[emmm202216269-bib-0060] Howard G , Goff DC (2012) Population shifts and the future of stroke: forecasts of the future burden of stroke. Ann N Y Acad Sci 1268: 14–20 22994216 10.1111/j.1749-6632.2012.06665.x PMC3727892

[emmm202216269-bib-0061] Hoyer FF , Naxerova K , Schloss MJ , Hulsmans M , Nair AV , Dutta P , Calcagno DM , Herisson F , Anzai A , Sun Y *et al* (2019) Tissue‐specific macrophage responses to remote injury impact the outcome of subsequent local immune challenge. Immunity 51: 899–914 31732166 10.1016/j.immuni.2019.10.010 PMC6892583

[emmm202216269-bib-0062] Iadecola C , Buckwalter MS , Anrather J (2020) Immune responses to stroke: mechanisms, modulation, and therapeutic potential. J Clin Investig 130: 2777–2788 32391806 10.1172/JCI135530 PMC7260029

[emmm202216269-bib-0063] Jager J , Grémeaux T , Cormont M , Le Marchand‐Brustel Y , Tanti J‐F (2007) Interleukin‐1β‐induced insulin resistance in adipocytes through down‐regulation of insulin receptor substrate‐1 expression. Endocrinology 148: 241–251 17038556 10.1210/en.2006-0692 PMC1971114

[emmm202216269-bib-0064] Jiang C , Kong W , Wang Y , Ziai W , Yang Q , Zuo F , Li F , Wang Y , Xu H , Li Q *et al* (2017) Changes in the cellular immune system and circulating inflammatory markers of stroke patients. Oncotarget 8: 3553–3567 27682880 10.18632/oncotarget.12201 PMC5356903

[emmm202216269-bib-0065] Kallmünzer B , Breuer L , Kahl N , Bobinger T , Raaz‐Schrauder D , Huttner HB , Schwab S , Köhrmann M (2012) Serious cardiac arrhythmias after stroke: incidence, time course, and predictors‐a systematic, prospective analysis. Stroke 43: 2892–2897 22961962 10.1161/STROKEAHA.112.664318

[emmm202216269-bib-0066] Kang R , Chen R , Zhang Q , Hou W , Wu S , Cao L , Huang J , Yu Y , Fan X , Yan Z *et al* (2014) HMGB1 in health and disease. Mol Aspects Med 40: 1–116 25010388 10.1016/j.mam.2014.05.001 PMC4254084

[emmm202216269-bib-0067] Kaptoge S , Seshasai SRK , Gao P , Freitag DF , Butterworth AS , Borglykke A , Di Angelantonio E , Gudnason V , Rumley A , Lowe GDO *et al* (2014) Inflammatory cytokines and risk of coronary heart disease: new prospective study and updated meta‐analysis. Eur Heart J 35: 578–589 24026779 10.1093/eurheartj/eht367 PMC3938862

[emmm202216269-bib-0068] Katan M , Luft A (2018) Global burden of stroke. Semin Neurol 38: 208–211 29791947 10.1055/s-0038-1649503

[emmm202216269-bib-0069] Katsanos AH , Palaiodimou L , Price C , Giannopoulos S , Lemmens R , Kosmidou M , Georgakis MK , Weimar C , Kelly PJ , Tsivgoulis G (2020) Colchicine for stroke prevention in patients with coronary artery disease: a systematic review and meta‐analysis. Eur J Neurol 27: 1035–1038 32134555 10.1111/ene.14198

[emmm202216269-bib-0070] Kelly P , Weimar C , Lemmens R , Murphy S , Purroy F , Arsovska A , Bornstein NM , Czlonkowska A , Fischer U , Fonseca AC *et al* (2021) Colchicine for prevention of vascular inflammation in Non‐CardioEmbolic stroke (CONVINCE) – study protocol for a randomised controlled trial. Eur Stroke J 6: 222–228 34414298 10.1177/2396987320972566 PMC8370082

[emmm202216269-bib-0071] Kernan WN , Ovbiagele B , Black HR , Bravata DM , Chimowitz MI , Ezekowitz MD , Fang MC , Fisher M , Furie KL , Heck DV *et al* (2014) Guidelines for the prevention of stroke in patients with stroke and transient ischemic attack: a guideline for healthcare professionals from the American Heart Association/American Stroke Association. Stroke 45: 2160–2236 24788967 10.1161/STR.0000000000000024

[emmm202216269-bib-0072] Kim W , Kim EJ (2018) Heart failure as a risk factor for stroke. J Stroke 20: 33–45 29402070 10.5853/jos.2017.02810 PMC5836579

[emmm202216269-bib-0073] Kim JB , Joon SC , Yu YM , Nam K , Piao CS , Kim SW , Lee MH , Han PL , Park JS , Lee JK (2006) HMGB1, a novel cytokine‐like mediator linking acute neuronal death and delayed neuroinflammation in the postischemic brain. J Neurosci 26: 6413–6421 16775128 10.1523/JNEUROSCI.3815-05.2006 PMC6674036

[emmm202216269-bib-0074] Kim I‐D , Lee H , Kim S‐W , Lee H‐K , Choi J , Han P‐L , Lee J‐K (2018) Alarmin HMGB1 induces systemic and brain inflammatory exacerbation in post‐stroke infection rat model. Cell Death Dis 9: 426 29555931 10.1038/s41419-018-0438-8 PMC5859283

[emmm202216269-bib-0075] Kim SW , Lee H , Lee HK , Kim ID , Lee JK (2019) Neutrophil extracellular trap induced by HMGB1 exacerbates damages in the ischemic brain. Acta Neuropathol Commun 7: 94 31177989 10.1186/s40478-019-0747-x PMC6556959

[emmm202216269-bib-0076] Kirchgesner J , Beaugerie L , Carrat F , Andersen NN , Jess T , Schwarzinger M , Bouvier AM , Buisson A , Carbonnel F , Cosnes J *et al* (2018) Increased risk of acute arterial events in young patients and severely active IBD: a nationwide French cohort study. Gut 67: 1261–1268 28647686 10.1136/gutjnl-2017-314015

[emmm202216269-bib-0077] Klehmet J , Harms H , Richter M , Prass K , Volk HD , Dirnagl U , Meisel A , Meisel C (2009) Stroke‐induced immunodepression and post‐stroke infections: lessons from the preventive antibacterial therapy in stroke trial. Neuroscience 158: 1184–1193 18722511 10.1016/j.neuroscience.2008.07.044

[emmm202216269-bib-0078] Krishnan S , O'Boyle C , Smith CJ , Hulme S , Allan SM , Grainger JR , Lawrence CB (2021) A hyperacute immune map of ischaemic stroke patients reveals alterations to circulating innate and adaptive cells. Clin Exp Immunol 203: 458–471 33205448 10.1111/cei.13551 PMC7874838

[emmm202216269-bib-0079] Kunze A , Zierath D , Tanzi P , Cain K , Becker K (2014) Peroxiredoxin 5 (PRX5) is correlated inversely to systemic markers of inflammation in acute stroke. Stroke 45: 608–610 24385276 10.1161/STROKEAHA.113.003813 PMC3946812

[emmm202216269-bib-0080] Ladenvall C , Jood K , Blomstrand C , Nilsson S , Jern C , Ladenvall P (2006) Serum C‐reactive protein concentration and genotype in relation to ischemic stroke subtype. Stroke 37: 2018–2023 16809555 10.1161/01.STR.0000231872.86071.68

[emmm202216269-bib-0081] Lassarén P , Lindblad C , Frostell A , Carpenter KLH , Guilfoyle MR , Hutchinson PJA , Helmy A , Thelin EP (2021) Systemic inflammation alters the neuroinflammatory response: a prospective clinical trial in traumatic brain injury. J Neuroinflammation 18: 221 34563211 10.1186/s12974-021-02264-2 PMC8464153

[emmm202216269-bib-0082] Lau LH , Lew J , Borschmann K , Thijs V , Ekinci EI (2019) Prevalence of diabetes and its effects on stroke outcomes: a meta‐analysis and literature review. J Diabetes Investig 10: 780–792 10.1111/jdi.12932 PMC6497593 30220102

[emmm202216269-bib-0083] Lee J , D'Aigle J , Atadja L , Quaicoe V , Honarpisheh P , Ganesh BP , Hassan A , Graf J , Petrosino J , Putluri N *et al* (2020) Gut microbiota–derived short‐chain fatty acids promote poststroke recovery in aged mice. Circ Res 127: 453–465 32354259 10.1161/CIRCRESAHA.119.316448 PMC7415518

[emmm202216269-bib-0084] Li D , Wu M (2021) Pattern recognition receptors in health and diseases. Signal Transduct Target Ther 6: 291 34344870 10.1038/s41392-021-00687-0 PMC8333067

[emmm202216269-bib-0085] Li WA , Moore‐Langston S , Chakraborty T , Rafols JA , Conti AC , Ding Y (2013) Hyperglycemia in stroke and possible treatments. Neurol Res 35: 479–491 23622737 10.1179/1743132813Y.0000000209

[emmm202216269-bib-0086] Li Y , Zhu Z , Huang T , Zhou Y , Wang X , Yang L , Chen Z , Yu W , Li P (2018) The peripheral immune response after stroke—a double edge sword for blood‐brain barrier integrity. CNS Neurosci Ther 24: 1115–1128 30387323 10.1111/cns.13081 PMC6490160

[emmm202216269-bib-0087] Liesz A , Rüger H , Purrucker J , Zorn M , Dalpke A , Möhlenbruch M , Englert S , Nawroth PP , Veltkamp R (2013) Stress mediators and immune dysfunction in patients with acute cerebrovascular diseases. PLoS One 8: 1–10 10.1371/journal.pone.0074839 PMC3777986 24069356

[emmm202216269-bib-0088] Liesz A , Dalpke A , Mracsko E , Antoine DJ , Roth S , Zhou W , Yang H , Na SY , Akhisaroglu M , Fleming T *et al* (2015) DAMP signaling is a key pathway inducing immune modulation after brain injury. J Neurosci 35: 583–598 25589753 10.1523/JNEUROSCI.2439-14.2015 PMC4293412

[emmm202216269-bib-0089] Liu Q , Johnson EM , Lam RK , Wang Q , Bo Ye H , Wilson EN , Minhas PS , Liu L , Swarovski MS , Tran S *et al* (2019) Peripheral TREM1 responses to brain and intestinal immunogens amplify stroke severity. Nat Immunol 20: 1023–1034 31263278 10.1038/s41590-019-0421-2 PMC6778967

[emmm202216269-bib-0090] Lin HB , Wei GS , Li FX , Guo WJ , Hong P , Weng YQ , Zhang QQ , Xu SY , Bin LW , You ZJ *et al* (2020) Macrophage–NLRP3 inflammasome activation exacerbates cardiac dysfunction after ischemic stroke in a mouse model of diabetes. Neurosci Bull 36: 1035–1045 32683554 10.1007/s12264-020-00544-0 PMC7475163

[emmm202216269-bib-0091] Luitse MJA , Biessels GJ , Rutten GEHM , Kappelle LJ (2012) Diabetes, hyperglycaemia, and acute ischaemic stroke. Lancet Neurol 11: 261–271 22341034 10.1016/S1474-4422(12)70005-4

[emmm202216269-bib-0092] Lv T , Zhao B , Hu Q , Zhang X (2021) The glymphatic system: a novel therapeutic target for stroke treatment. Front Aging Neurosci 13: 1–16 10.3389/fnagi.2021.689098 PMC8297504 34305569

[emmm202216269-bib-0093] Maier IL , Karch A , Mikolajczyk R , Bähr M , Liman J (2015) Effect of beta‐blocker therapy on the risk of infections and death after acute stroke – a historical cohort study. PLoS One 10: 1–10 10.1371/journal.pone.0116836 PMC4314079 25643360

[emmm202216269-bib-0094] Maier IL , Becker JC , Leyhe JR , Schnieder M , Behme D , Psychogios MN , Liman J (2018) Influence of beta‐blocker therapy on the risk of infections and death in patients at high risk for stroke induced immunodepression. PLoS One 13: 1–11 10.1371/journal.pone.0196174 PMC5919008 29694433

[emmm202216269-bib-0095] Mann DL (2015) Innate immunity and the failing heart. Circ Res 116: 1254–1268 25814686 10.1161/CIRCRESAHA.116.302317 PMC4380242

[emmm202216269-bib-0096] Martínez GJ , Celermajer DS , Patel S (2018) The NLRP3 inflammasome and the emerging role of colchicine to inhibit atherosclerosis‐associated inflammation. Atherosclerosis 269: 262–271 29352570 10.1016/j.atherosclerosis.2017.12.027

[emmm202216269-bib-0097] Masson W , Lobo M , Molinero G , Masson G , Lavalle‐Cobo A (2020) Role of colchicine in stroke prevention: an updated meta‐analysis. J Stroke Cerebrovasc Dis 29: 104756 32160956 10.1016/j.jstrokecerebrovasdis.2020.104756

[emmm202216269-bib-0098] McCulloch L , Alfieri A , McColl BW (2018) Experimental stroke differentially affects discrete subpopulations of splenic macrophages. Front Immunol 9: 1108 29872438 10.3389/fimmu.2018.01108 PMC5972287

[emmm202216269-bib-0099] Meloux A , Rigal E , Rochette L , Cottin Y , Bejot Y , Vergely C (2018) Ischemic stroke increases heart vulnerability to ischemia‐reperfusion and alters myocardial cardioprotective pathways. Stroke 49: 2752–2760 30355197 10.1161/STROKEAHA.118.022207

[emmm202216269-bib-0100] Moschonas IC , Tselepis AD (2019) The pathway of neutrophil extracellular traps towards atherosclerosis and thrombosis. Atherosclerosis 288: 9–16 31280097 10.1016/j.atherosclerosis.2019.06.919

[emmm202216269-bib-0101] Mracsko E , Liesz A , Karcher S , Zorn M , Bari F , Veltkamp R (2014) Differential effects of sympathetic nervous system and hypothalamic‐pituitary‐adrenal axis on systemic immune cells after severe experimental stroke. Brain Behav Immun 41: 200–209 24886966 10.1016/j.bbi.2014.05.015

[emmm202216269-bib-0102] Muhammad S , Barakat W , Stoyanov S , Murikinati S , Yang H , Tracey KJ , Bendszus M , Rossetti G , Nawroth PP , Bierhaus A *et al* (2008) The HMGB1 receptor RAGE mediates ischemic brain damage. J Neurosci 28: 12023–12031 19005067 10.1523/JNEUROSCI.2435-08.2008 PMC4597312

[emmm202216269-bib-0103] Muller LMAJ , Gorter KJ , Hak E , Goudzwaard WL , Schellevis FG , Hoepelman AIM , Rutten GEHM (2005) Increased risk of common infections in patients with type 1 and type 2 diabetes mellitus. Clin Infect Dis 41: 281–288 16007521 10.1086/431587

[emmm202216269-bib-0104] Nidorf SM , Eikelboom JW , Budgeon CA , Thompson PL (2013) Low‐dose colchicine for secondary prevention of cardiovascular disease. J Am Coll Cardiol 61: 404–410 23265346 10.1016/j.jacc.2012.10.027

[emmm202216269-bib-0105] Norberg E , Odenstedt‐Herges H , Rydenhag B , Oras J (2018) Impact of acute cardiac complications after subarachnoid hemorrhage on long‐term mortality and cardiovascular events. Neurocrit Care 29: 404–412 29949009 10.1007/s12028-018-0558-0 PMC6290719

[emmm202216269-bib-0106] O'Connell GC , Petrone AB , Tennant CS , Lucke‐Wold N , Kabbani Y , Tarabishy AR , Chantler PD , Barr TL (2017) Circulating extracellular DNA levels are acutely elevated in ischaemic stroke and associated with innate immune system activation. Brain Inj 31: 1369–1375 28585898 10.1080/02699052.2017.1312018

[emmm202216269-bib-0107] Offner H , Subramanian S , Parker SM , Afentoulis ME , Vandenbark AA , Hurn PD (2006a) Experimental stroke induces massive, rapid activation of the peripheral immune system. J Cereb Blood Flow Metab 26: 654–665 16121126 10.1038/sj.jcbfm.9600217

[emmm202216269-bib-0108] Offner H , Subramanian S , Parker SM , Wang C , Afentoulis ME , Lewis A , Vandenbark AA , Hurn PD (2006b) Splenic atrophy in experimental stroke is accompanied by increased regulatory T cells and circulating macrophages. J Immunol 176: 6523–6531 16709809 10.4049/jimmunol.176.11.6523

[emmm202216269-bib-0109] Oras J , Grivans C , Bartley A , Rydenhag B , Ricksten SE , Seeman‐Lodding H (2016) Elevated high‐sensitive troponin T on admission is an indicator of poor long‐term outcome in patients with subarachnoid haemorrhage: a prospective observational study. Crit Care 20: 1–10 26781032 10.1186/s13054-015-1181-5 PMC4717610

[emmm202216269-bib-0110] Parish S , Arnold M , Clarke R , Du H , Wan E , Kurmi O , Chen Y , Guo Y , Bian Z , Collins R *et al* (2019) Assessment of the role of carotid atherosclerosis in the association between major cardiovascular risk factors and ischemic stroke subtypes. JAMA Netw Open 2: 5 10.1001/jamanetworkopen.2019.4873 PMC6547114 31150080

[emmm202216269-bib-0111] Patel RAG , McMullen PW (2017) Neuroprotection in the treatment of acute ischemic stroke. Prog Cardiovasc Dis 59: 542–548 28465001 10.1016/j.pcad.2017.04.005

[emmm202216269-bib-0112] Planas AM , Gomez‐Choco M , Urra X , Gorina R , Caballero M , Chamorro A (2012) Brain‐derived antigens in lymphoid tissue of patients with acute stroke. J Immunol 188: 2156–2163 22287710 10.4049/jimmunol.1102289

[emmm202216269-bib-0113] Prabhu SD (2004) Cytokine‐induced modulation of cardiac function. Circ Res 95: 1140–1153 15591236 10.1161/01.RES.0000150734.79804.92

[emmm202216269-bib-0114] Prass K , Meisel C , Höflich C , Braun J , Halle E , Wolf T , Ruscher K , Victorov IV , Priller J , Dirnagl U *et al* (2003) Stroke‐induced immunodeficiency promotes spontaneous bacterial infections and is mediated by sympathetic activation reversal by poststroke T helper cell type 1–like immunostimulation. J Exp Med 198: 725–736 12939340 10.1084/jem.20021098 PMC2194193

[emmm202216269-bib-0115] Prosser J , MacGregor L , Lees KR , Diener HC , Hacke W , Davis S (2007) Predictors of early cardiac morbidity and mortality after ischemic stroke. Stroke 38: 2295–2302 17569877 10.1161/STROKEAHA.106.471813

[emmm202216269-bib-0116] Rasmussen MK , Mestre H , Nedergaard M (2018) The glymphatic pathway in neurological disorders. Lancet Neurol 17: 1016–1024 30353860 10.1016/S1474-4422(18)30318-1 PMC6261373

[emmm202216269-bib-0117] Richard S , Lapierre V , Girerd N , Bonnerot M , Burkhard PR , Lagerstedt L , Bracard S , Debouverie M , Turck N , Sanchez JC (2016) Diagnostic performance of peroxiredoxin 1 to determine time‐of‐onset of acute cerebral infarction. Sci Rep 6: 1–10 27924073 10.1038/srep38300 PMC5141372

[emmm202216269-bib-0118] Ridker PM , Everett BM , Thuren T , MacFadyen JG , Chang WH , Ballantyne C , Fonseca F , Nicolau J , Koenig W , Anker SD *et al* (2017) Antiinflammatory therapy with canakinumab for atherosclerotic disease. N Engl J Med 377: 1119–1131 28845751 10.1056/NEJMoa1707914

[emmm202216269-bib-0119] Rosas‐Ballina M , Tracey KJ (2009) Cholinergic control of inflammation. J Intern Med 265: 663–679 19493060 10.1111/j.1365-2796.2009.02098.x PMC4540232

[emmm202216269-bib-0120] Rosas‐Ballina M , Olofsson PS , Ochani M , Valdés‐Ferrer SI , Levine YA , Reardon C , Tusche MW , Pavlov VA , Andersson U , Chavan S *et al* (2011) Acetylcholine‐synthesizing T cells relay neural signals in a vagus nerve circuit. Science 334: 98–101 21921156 10.1126/science.1209985 PMC4548937

[emmm202216269-bib-0121] Roth S , Singh V , Tiedt S , Schindler L , Huber G , Geerlof A , Antoine DJ , Anfray A , Orset C , Gauberti M *et al* (2018) Brain‐released alarmins and stress response synergize in accelerating atherosclerosis progression after stroke. Sci Transl Med 10: 1–12 10.1126/scitranslmed.aao1313 29540615

[emmm202216269-bib-0122] Roth S , Cao J , Singh V , Tiedt S , Hundeshagen G , Li T , Boehme JD , Chauhan D , Zhu J , Ricci A *et al* (2021a) Post‐injury immunosuppression and secondary infections are caused by an AIM2 inflammasome‐driven signaling cascade. Immunity 54: 648–659 33667383 10.1016/j.immuni.2021.02.004

[emmm202216269-bib-0123] Roth S , Yang J , Cramer JV , Malik R , Liesz A (2021b) Detection of cytokine‐induced sickness behavior after ischemic stroke by an optimized behavioral assessment battery. Brain Behav Immun 91: 668–672 33197540 10.1016/j.bbi.2020.11.016

[emmm202216269-bib-0124] Ruscitti P , Masedu F , Alvaro S , Airò P , Battafarano N , Cantarini L , Cantatore FP , Carlino G , D'Abrosca V , Frassi M *et al* (2019) Anti‐interleukin‐1 treatment in patients with rheumatoid arthritis and type 2 diabetes (TRACK): a multicentre, open‐label, randomised controlled trial. PLoS Med 16: 1–22 10.1371/journal.pmed.1002901 PMC6742232 31513665

[emmm202216269-bib-0125] Ruthirago D , Julayanont P , Tantrachoti P , Kim J , Nugent K (2016) Cardiac arrhythmias and abnormal electrocardiograms after acute stroke. Am J Med Sci 351: 112–118 26802767 10.1016/j.amjms.2015.10.020

[emmm202216269-bib-0126] Salzano S , Checconi P , Hanschmann EM , Lillig CH , Bowler LD , Chan P , Vaudry D , Mengozzi M , Coppo L , Sacre S *et al* (2014) Linkage of inflammation and oxidative stress via release of glutathionylated peroxiredoxin‐2, which acts as a danger signal. Proc Natl Acad Sci USA 111: 12157–12162 25097261 10.1073/pnas.1401712111 PMC4143057

[emmm202216269-bib-0127] Schaeffer S , Iadecola C (2021) Revisiting the neurovascular unit. Nat Neurosci 24: 1198–1209 34354283 10.1038/s41593-021-00904-7 PMC9462551

[emmm202216269-bib-0128] Schuetz P , Castro P , Shapiro NI (2011) Diabetes and sepsis: preclinical findings and clinical relevance. Diabetes Care 34: 771–778 21357364 10.2337/dc10-1185 PMC3041224

[emmm202216269-bib-0129] Schuhmann MK , Kollikowski AM , März AG , Bieber M , Pham M , Stoll G (2021) Danger‐associated molecular patterns are locally released during occlusion in hyper‐acute stroke. Brain Behav Immun Health 15: 100270 34589775 10.1016/j.bbih.2021.100270 PMC8474429

[emmm202216269-bib-0130] Schulze J , Zierath D , Tanzi P , Cain K , Shibata D , Dressel A , Becker K (2013) Severe stroke induces long‐lasting alterations of high‐mobility group box 1. Stroke 44: 246–248 23204053 10.1161/STROKEAHA.112.676072 PMC3530419

[emmm202216269-bib-0131] Seifert HA , Hall AA , Chapman CB , Collier LA , Willing AE , Pennypacker KR (2012) A transient decrease in spleen size following stroke corresponds to splenocyte release into systemic circulation. J Neuroimmune Pharmacol 7: 1017–1024 23054371 10.1007/s11481-012-9406-8 PMC3518577

[emmm202216269-bib-0132] Sharma A , Tate M , Mathew G , Vince JE , Ritchie RH , De Haan JB (2018) Oxidative stress and NLRP3‐inflammasome activity as significant drivers of diabetic cardiovascular complications: therapeutic implications. Front Physiol 9: 1–15 29515457 10.3389/fphys.2018.00114 PMC5826188

[emmm202216269-bib-0133] Singh V , Roth S , Llovera G , Sadler R , Garzetti D , Stecher B , Dichgans M , Liesz A (2016) Microbiota dysbiosis controls the neuroinflammatory response after stroke. J Neurosci 36: 7428–7440 27413153 10.1523/JNEUROSCI.1114-16.2016 PMC6705544

[emmm202216269-bib-0134] Sippel TR , Shimizu T , Strnad F , Traystman RJ , Herson PS , Waziri A (2015) Arginase I release from activated neutrophils induces peripheral immunosuppression in a murine model of stroke. J Cereb Blood Flow Metab 35: 1657–1663 25966956 10.1038/jcbfm.2015.103 PMC4640306

[emmm202216269-bib-0135] Smith CJ , Hulme S , Vail A , Heal C , Parry‐Jones AR , Scarth S , Hopkins K , Hoadley M , Allan SM , Rothwell NJ *et al* (2018) SCIL‐STROKE (Subcutaneous Interleukin‐1 Receptor Antagonist in Ischemic Stroke). Stroke 49: 1210–1216 29567761 10.1161/STROKEAHA.118.020750

[emmm202216269-bib-0136] Stanley D , Mason LJ , MacKin KE , Srikhanta YN , Lyras D , Prakash MD , Nurgali K , Venegas A , Hill MD , Moore RJ *et al* (2016) Translocation and dissemination of commensal bacteria in post‐stroke infection. Nat Med 22: 1277–1284 27694934 10.1038/nm.4194

[emmm202216269-bib-0137] Stanne TM , Angerfors A , Andersson B , Brännmark C , Holmegaard L , Jern C (2022) Longitudinal study reveals long‐term proinflammatory proteomic signature after ischemic stroke across subtypes. Stroke: 1–12 10.1161/STROKEAHA.121.038349 PMC9389938 35686557

[emmm202216269-bib-0138] Stark K , Massberg S (2021) Interplay between inflammation and thrombosis in cardiovascular pathology. Nat Rev Cardiol 18: 666–682 33958774 10.1038/s41569-021-00552-1 PMC8100938

[emmm202216269-bib-0139] Suissa L , Panicucci E , Perot C , Romero G , Gazzola S , Laksiri N , Rey C , Doche E , Mahagne M‐H , Pelletier J *et al* (2020) Effect of hyperglycemia on stroke outcome is not homogeneous to all patients treated with mechanical thrombectomy. Clin Neurol Neurosurg 194: 105750 32248045 10.1016/j.clineuro.2020.105750

[emmm202216269-bib-0140] Sun L , Ai J , Wang N , Zhang R , Li J , Zhang T , Wu W , Hang P , Lu Y , Yang B (2010) Cerebral ischemia elicits aberration in myocardium contractile function and intracellular calcium handling. Cell Physiol Biochem 26: 421–430 20798527 10.1159/000320584

[emmm202216269-bib-0141] Swidsinski A , Loening‐Baucke V , Krüger M , Kirsch S (2012) Central nervous system and the colonic bioreactor: analysis of colonic microbiota in patients with stroke unravels unknown mechanisms of the host defense after brain injury. Intest Res 10: 332

[emmm202216269-bib-0142] Sykora M , Siarnik P , Diedler J (2015) β‐blockers, pneumonia, and outcome after ischemic stroke. Stroke 46: 1269–1274 25899243 10.1161/STROKEAHA.114.008260

[emmm202216269-bib-0143] Tardif J‐C , Kouz S , Waters DD , Bertrand OF , Diaz R , Maggioni AP , Pinto FJ , Ibrahim R , Gamra H , Kiwan GS *et al* (2019) Efficacy and safety of low‐dose colchicine after myocardial infarction. N Engl J Med 381: 2497–2505 31733140 10.1056/NEJMoa1912388

[emmm202216269-bib-0144] Tsai NW , Lin TK , Der CS , Chang WN , Wang HC , Yang TM , Lin YJ , Jan CR , Huang CR , Liou CW *et al* (2011) The value of serial plasma nuclear and mitochondrial DNA levels in patients with acute ischemic stroke. Clin Chim Acta 412: 476–479 21130757 10.1016/j.cca.2010.11.036

[emmm202216269-bib-0145] Tsalamandris S , Antonopoulos AS , Oikonomou E , Papamikroulis G , Vogiatzi G (2019) Risk factors and cardiovascular disease prevention the role of inflammation in diabetes: current concepts and future perspectives. Eur Cardiol Rev 14: 50–59 10.15420/ecr.2018.33.1 PMC6523054 31131037

[emmm202216269-bib-0146] Tsukagawa T , Katsumata R , Fujita M , Yasui K , Akhoon C , Ono K , Dohi K , Aruga T (2017) Elevated serum high‐mobility group box‐1 protein level is associated with poor functional outcome in ischemic stroke. J Stroke Cerebrovasc Dis 26: 2404–2411 28645523 10.1016/j.jstrokecerebrovasdis.2017.05.033

[emmm202216269-bib-0147] Tuttolomondo A , Di Sciacca R , Di Raimondo D , Serio A , D'Aguanno G , La Placa S , Pecoraro R , Arnao V , Marino L , Monaco S *et al* (2009) Plasma levels of inflammatory and thrombotic/fibrinolytic markers in acute ischemic strokes: relationship with TOAST subtype, outcome and infarct site. J Neuroimmunol 215: 84–89 19695716 10.1016/j.jneuroim.2009.06.019

[emmm202216269-bib-0148] Urra X , Cervera Á , Obach V , Climent N , Planas AM , Chamorro A (2009) Monocytes are major players in the prognosis and risk of infection after acute stroke. Stroke 40: 1262–1268 19164783 10.1161/STROKEAHA.108.532085

[emmm202216269-bib-0149] Vallés J , Lago A , Santos MT , Latorre AM , Tembl JI , Salom JB , Nieves C , Moscardó A (2017) Neutrophil extracellular traps are increased in patients with acute ischemic stroke: prognostic significance. Thromb Haemost 117: 1919–1929 28837206 10.1160/TH17-02-0130

[emmm202216269-bib-0150] Vandanmagsar B , Youm YH , Ravussin A , Galgani JE , Stadler K , Mynatt RL , Ravussin E , Stephens JM , Dixit VD (2011) The NLRP3 inflammasome instigates obesity‐induced inflammation and insulin resistance. Nat Med 17: 179–189 21217695 10.1038/nm.2279 PMC3076025

[emmm202216269-bib-0151] Vedantam A , Brennan J , Levin HS , McCarthy JJ , Dash PK , Redell JB , Yamal JM , Robertson CS (2021) Early versus late profiles of inflammatory cytokines after mild traumatic brain injury and their association with neuropsychological outcomes. J Neurotrauma 38: 53–62 32600167 10.1089/neu.2019.6979 PMC7757539

[emmm202216269-bib-0152] Veltkamp R , Uhlmann S , Marinescu M , Sticht C , Finke D , Gretz N , Gröne HJ , Katus HA , Backs J , Lehmann LH (2019) Experimental ischaemic stroke induces transient cardiac atrophy and dysfunction. J Cachexia Sarcopenia Muscle 10: 54–62 30378296 10.1002/jcsm.12335 PMC6438414

[emmm202216269-bib-0153] Verma G , Bhatia H , Datta M (2013) JNK1/2 regulates ER‐mitochondrial Ca^2+^ cross‐talk during IL‐1β‐mediated cell death in RINm5F and human primary β‐cells. Mol Biol Cell 24: 2058–2071 23615449 10.1091/mbc.E12-12-0885 PMC3681707

[emmm202216269-bib-0154] Vermeij JD , Westendorp WF , Dippel DWJ , van de Beek D , Nederkoorn PJ (2018) Antibiotic therapy for preventing infections in people with acute stroke. Cochrane Database Syst Rev 2018: CD008530 10.1002/14651858.CD008530.pub3 PMC6491314 29355906

[emmm202216269-bib-0155] Virani SS , Alonso A , Aparicio HJ , Benjamin EJ , Bittencourt MS , Callaway CW , Carson AP , Chamberlain AM , Cheng S , Delling FN *et al* (2021) Heart disease and stroke statistics—2021 update. Circulation 143: e254–e743 33501848 10.1161/CIR.0000000000000950 PMC13036842

[emmm202216269-bib-0156] Waje‐Andreassen U , Kråkenes J , Ulvestad E , Thomassen L , Myhr K‐M , Aarseth J , Vedeler CA (2005) IL‐6: an early marker for outcome in acute ischemic stroke. Acta Neurol Scand 111: 360–365 15876336 10.1111/j.1600-0404.2005.00416.x

[emmm202216269-bib-0157] Wang J , Yu L , Jiang C , Fu X , Liu X , Wang M , Ou C , Cui X , Zhou C , Wang J (2015) Cerebral ischemia increases bone marrow CD4^+^CD25^+^FoxP3^+^ regulatory T cells in mice via signals from sympathetic nervous system. Brain Behav Immun 43: 172–183 25110149 10.1016/j.bbi.2014.07.022 PMC4258426

[emmm202216269-bib-0158] Wang Y , Qian Y , Smerin D , Zhang S , Zhao Q , Xiong X (2019) Newly detected atrial fibrillation after acute stroke: a narrative review of causes and implications. Cardiology 144: 112–121 31600748 10.1159/000502971

[emmm202216269-bib-0159] Warnatsch A , Ioannou M , Wang Q , Papayannopoulos V (2015) Neutrophil extracellular traps license macrophages for cytokine production in atherosclerosis. Science 349: 316–320 26185250 10.1126/science.aaa8064 PMC4854322

[emmm202216269-bib-0160] Wartenberg KE , Stoll A , Funk A , Meyer A , Schmidt JM , Berrouschot J (2011) Infection after acute ischemic stroke: risk factors, biomarkers, and outcome. Stroke Res Treat 2011: 1–8 10.4061/2011/830614 PMC3140159 21789273

[emmm202216269-bib-0161] Wen H , Gris D , Lei Y , Jha S , Zhang L , Huang MTH , Brickey WJ , Ting JPY (2011) Fatty acid‐induced NLRP3‐ASC inflammasome activation interferes with insulin signaling. Nat Immunol 12: 408–415 21478880 10.1038/ni.2022 PMC4090391

[emmm202216269-bib-0162] Westendorp WF , Nederkoorn PJ , Vermeij J‐D , Dijkgraaf MG & de Beek D van (2011) Post‐stroke infection: a systematic review and meta‐analysis. BMC Neurol 11: 110 21933425 10.1186/1471-2377-11-110 PMC3185266

[emmm202216269-bib-0163] Westendorp WF , Vermeij J‐D , Brouwer MC , Roos YBWEM , Nederkoorn PJ , van de Beek D (2016) Pre‐stroke use of beta‐blockers does not lower post‐stroke infection rate: an exploratory analysis of the preventive antibiotics in stroke study. Cerebrovasc Dis 42: 506–511 27701170 10.1159/000450926 PMC5296919

[emmm202216269-bib-0164] Willerson JT , Ridker PM (2004) Inflammation as a cardiovascular risk factor. Circulation 109: 2–10 15173056 10.1161/01.CIR.0000129535.04194.38

[emmm202216269-bib-0165] Williams LS , Rotich J , Qi R , Fineberg N , Espay A , Bruno A , Fineberg SE , Tierney WR (2002) Effects of admission hyperglycemia on mortality and costs in acute ischemic stroke. Neurology 59: 67–71 12105309 10.1212/wnl.59.1.67

[emmm202216269-bib-0166] Xu K , Gao X , Xia G , Chen M , Zeng N , Wang S , You C , Tian X , Di H , Tang W *et al* (2021) Rapid gut dysbiosis induced by stroke exacerbates brain infarction in turn. Gut 70: 1486–1494 10.1136/gutjnl-2020-323263 33558272

[emmm202216269-bib-0167] Yan FL , Zhang JH (2014) Role of the sympathetic nervous system and spleen in experimental stroke‐induced immunodepression. Med Sci Monit 20: 2489–2496 25434807 10.12659/MSM.890844 PMC4260620

[emmm202216269-bib-0168] Yang ZS , Lin NN , Li L , Li Y (2016) The effect of TNF inhibitors on cardiovascular events in psoriasis and psoriatic arthritis: an updated meta‐analysis. Clin Rev Allergy Immunol 51: 240–247 27300248 10.1007/s12016-016-8560-9

[emmm202216269-bib-0169] Yang C , Hawkins KE , Doré S , Candelario‐Jalil E (2019) Neuroinflammatory mechanisms of blood‐brain barrier damage in ischemic stroke. Am J Physiol Cell Physiol 316: C135–C153 30379577 10.1152/ajpcell.00136.2018 PMC6397344

[emmm202216269-bib-0170] Yin J , Liao SX , He Y , Wang S , Xia GH , Liu FT , Zhu JJ , You C , Chen Q , Zhou L *et al* (2015) Dysbiosis of gut microbiota with reduced trimethylamine‐n‐oxide level in patients with large‐artery atherosclerotic stroke or transient ischemic attack. J Am Heart Assoc 4: 1–12 10.1161/JAHA.115.002699 PMC4845212 26597155

[emmm202216269-bib-0171] Zabala A , Darsalia V , Holzmann MJ , Franzén S , Svensson AM , Eliasson B , Patrone C , Nyström T , Jonsson M (2020) Risk of first stroke in people with type 2 diabetes and its relation to glycaemic control: a nationwide observational study. Diabetes Obes Metab 22: 182–190 31576643 10.1111/dom.13885

[emmm202216269-bib-0172] Zaremba J , Losy J (2001) Early TNF‐α levels correlate with ischaemic stroke severity. Acta Neurol Scand 104: 288–295 11696023 10.1034/j.1600-0404.2001.00053.x

[emmm202216269-bib-0173] Zatterale F , Longo M , Naderi J , Raciti GA , Desiderio A , Miele C , Beguinot F (2020) Chronic adipose tissue inflammation linking obesity to insulin resistance and type 2 diabetes. Front Physiol 10: 1–20 10.3389/fphys.2019.01607 PMC7000657 32063863

[emmm202216269-bib-0174] Zhang L , Chopp M , Zhang Y , Xiong Y , Li C , Sadry N , Rhaleb I , Lu M , Zhang ZG (2016) Diabetes mellitus impairs cognitive function in middle‐aged rats and neurological recovery in middle‐aged rats after stroke. Stroke 47: 2112–2118 27387991 10.1161/STROKEAHA.115.012578 PMC4961558

